# Designing Soluble PROTACs: Strategies and Preliminary Guidelines

**DOI:** 10.1021/acs.jmedchem.2c00201

**Published:** 2022-04-25

**Authors:** Diego García Jiménez, Matteo Rossi Sebastiano, Maura Vallaro, Valentina Mileo, Daniela Pizzirani, Elisa Moretti, Giuseppe Ermondi, Giulia Caron

**Affiliations:** † Molecular Biotechnology and Health Sciences Department, CASSMedChem, University of Torino, Via Quarello 15, 10135 Torino, Italy; ‡ Global Research and Preclinical Development, Research Center, Chiesi Farmaceutici, Largo Belloli 11/a, 43122 Parma, Italy; § Emerging Science & Technology Unit, Research Center, Chiesi Farmaceutici, Largo Belloli 11/a, 43122 Parma, Italy

## Abstract

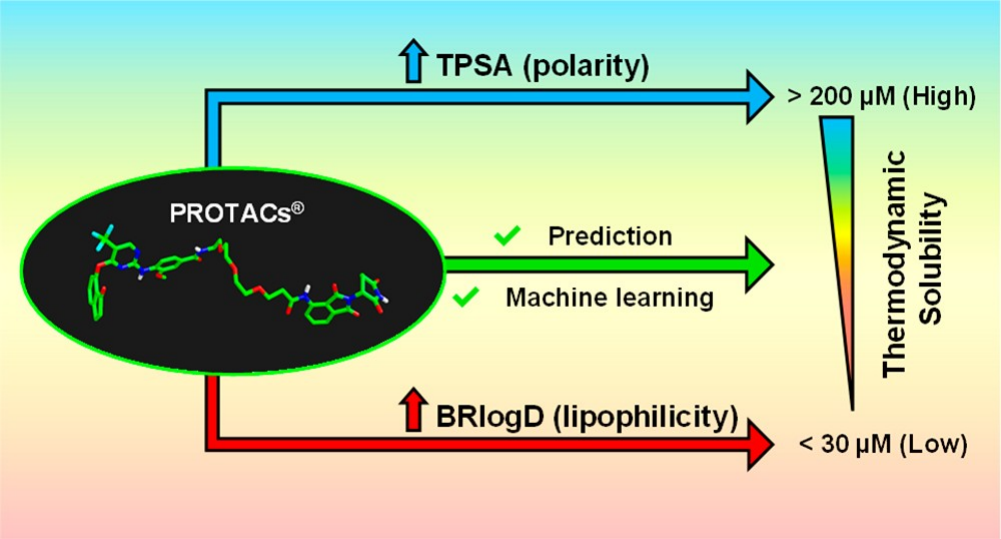

Solubility optimization is a crucial step to obtaining oral PROTACs. Here we measured the thermodynamic solubilities (log *S*) of 21 commercial PROTACs. Next, we measured BRlogD and log *k*_w_^IAM^ (lipophilicity), EPSA, and Δ log *k*_w_^IAM^ (polarity) and showed that lipophilicity plays a major role in governing log *S*, but a contribution of polarity cannot be neglected. Two-/three-dimensional descriptors calculated on conformers arising from conformational sampling and steered molecular dynamics failed in modeling solubility. Infographic tools were used to identify a privileged region of soluble PROTACs in a chemical space defined by BRlogD, log *k*_w_^IAM^ and topological polar surface area, while machine learning provided a log *S* classification model. Finally, for three pairs of PROTACs we measured the solubility, lipophilicity, and polarity of the building blocks and identified the limits of estimating PROTAC solubility from the synthetic components. Overall, this paper provides promising guidelines for optimizing PROTAC solubility in early drug discovery programs.

## Introduction

PROTACs are defined as heterobifunctional molecules built of three moieties or building blocks: a warhead binding a protein of interest (POI), an E3 ligase recruiter, and a linker attaching both regions. Indeed, since the first PROTAC was developed by Crews and Deshaies in 2001, their popularity in biomedical research and drug discovery has risen notably.^1^ This could be explained by their innovative mechanism of action that uses the degradative capacity of the proteasome to eliminate the desired target protein (TPD, targeted protein degradation).^2^ Thus, 2022 is witnessing the entry of novel degraders in clinical trials, with ARV-110 and ARV-471 already disclosed.^3^

PROTACs are widely known degraders and belong to the “beyond rule of 5” (bRo5) chemical space.^4^ Their large and flexible structure is responsible for drug metabolism and pharmacokinetics (DMPK) limitations that can hinder oral dosing.^5^ Therefore, it is crucial to study their *in vitro* ADME properties (solubility, permeability, etc.) to understand, monitor, and optimize their potential as oral drugs.^6^ Notably, at present, a well-established property-based drug design strategy is not available for this class of compounds.

Solubility (defined by the IUPAC as the “analytical composition of a saturated solution, expressed in terms of the proportion of a designated solute in a designated solvent”) has a crucial role in the success of any drug candidate.^7^ In fact, poor solubility can have an impact on various stages of the drug discovery process.^8^ Moreover, the interplay between solubility and permeability makes their simultaneous optimization a challenge for medicinal chemists.^9^ For example, increasing permeability by increasing lipophilicity may decrease solubility and metabolic stability.

It is widely believed that an acceptable solubility in the intestinal fluid is a prerequisite for achieving sufficiently high drug blood concentrations to obtain a systemic therapeutic effect. However, the definition of acceptable solubility is somewhat vague. In the early phase of discovery, where only aqueous solubility is of interest,^10^ it has been proposed that a good goal for solubility is >60 μg/mL.^11^ More recently some GSK researchers classified compounds into low (<30 μM), intermediate (30–200 μM), or highly soluble molecules (>200 μM) and applied these criteria in many internal drug discovery programs.^12^

Although solubility is a major issue in oncology programs where PROTACs are expected to be widely employed (compounds for treating cancer tend to have high doses), to our knowledge no specific report on PROTAC solubility has been reported up to now. A few papers about solubility in the bRo5 chemical space^13−15^ seem to suggest that the impact of the third dimension on solubility is less important than for cell permeability, but no data support this finding for degraders.^13^

To address the need for providing a strategy to optimize PROTAC solubility in drug discovery, here we set up a study focused on the determination of PROTAC experimental solubility and its main determinants. In particular, we focus on the following aims: (a) providing a data set of experimental aqueous thermodynamic solubility values and a pool of physicochemical descriptors (BRlogD, log *k*_w_^IAM^, Δ log *k*_w_^IAM^, etc.^16,17^) for a series of 21 commercial PROTACs, representative of the PROTAC chemical space; (b) evaluating the performance of common solubility prediction tools; (c) looking for the relationships between solubility and computed/experimental physicochemical descriptors; and (d) providing a classification system to be used in early drug discovery to distinguish soluble from not soluble degraders. Finally, we used three pairs of PROTACs to investigate the impact of the building blocks on the overall PROTAC solubility to evaluate how feasible modular prediction is.

Overall, this paper provides experimental data and preliminary guidelines to design soluble PROTACs. Notably this research is expected to be of utmost relevance within new chemical modalities, a current hot topic in medicinal chemistry and drug discovery.^18,19^

## Results and Discussion

### Experimental Solubility of the Considered Data Set

**Figure 1:**
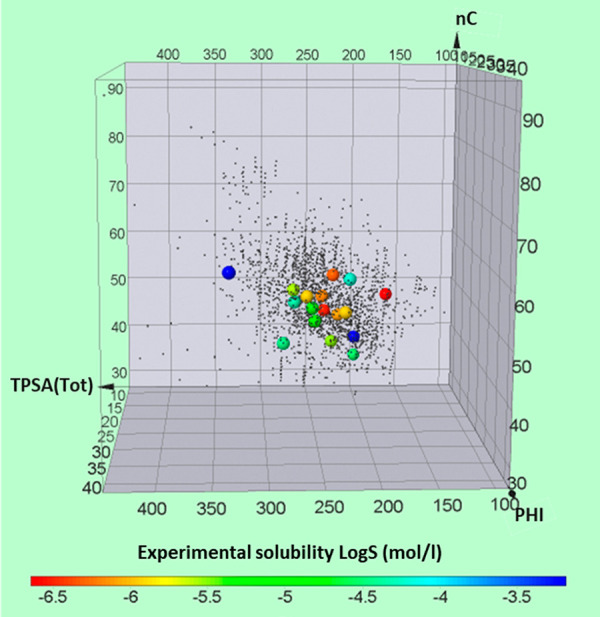
Graphical representation of the PROTAC solubility data set (21 commercial derivatives) based on 2D descriptors (nC, PHI, and TPSA). Large dots colors represent a solubility scale between −7 and −3 log *S* units (mol/L).

In previous papers we defined a PROTAC chemical space based on three representative 2D descriptors: the number of carbon atoms (nC), flexibility (PHI), and the topological polar surface area (TPSA) (Figure 1, PROTACs are the small black dots).^20,21^

With the use of this tool, 21 commercial degraders were selected with the aim to significantly represent this chemical space (large colored dots in Figure 1, structures in Figure S1). In fact, the PROTACs included in this study cover a substantial area of the defined descriptors: TPSA (166–335 Å^2^), nC (34–58), and PHI (9–27). Moreover, the PROTAC set is also structurally heterogeneous because of the presence of different E3 ligase ligands, linkers, and warheads (Table S1). For instance, 9 PROTACs use CRBN and 12 use VHL, the two major E3 ligase groups. Regarding the linker structure, alkyl, pegylated, and glycol moieties are the flexible linkers included in the investigated structures. One type of rigidifying linker (alkyne group) was also considered (MD-224). Moreover, the PROTAC set shows a wide variety of warheads. Finally, most of the considered PROTACs are predominantly neutral at pH 7.0 with some basic exceptions (Table S2).

In the pharmaceutical industry the type of solubility measurements is driven by the stage of the project, with kinetic solubility being the preferred method in early drug discovery.^22^ Its measurement only requires an initial DMSO stock solution which is precipitated by the addition of an aqueous phase.^23,24^ Here, we measured thermodynamic solubility, often determined in late lead optimization, which efficiently considers the solubility value when the equilibrium with the stable phase or polymorph has been reached. We decided to apply experimental conditions suitable for early drug discovery purposes: the shake-flask method with 1 h of incubation time at pH 7 (in order to obtain data comparable with chromatographic descriptors measured at pH 7) and 25 °C (a common temperature also allowing data comparison with Marvin calculated solubility). Solubility data are presented in Table 1 (accurate solubility values displayed as milligrams per milliliter and log *S* (mol/L) are available in Table S3) grouped by the GSK solubility classification: low (<30 μM), intermediate (30–200 μM), or highly soluble molecules (>200 μM).^7^ Results show that the three categories are significantly populated, although many degraders of the selected data set are poorly soluble. Solubilities for ACBI1, *cis*ACBI1, ARV-825, Mcl1 degrader-1, and MD-224 were below the quantification limit, and thus these five PROTACs were excluded from any quantitative analysis but were classified in the low solubility group.

**Table 1:**
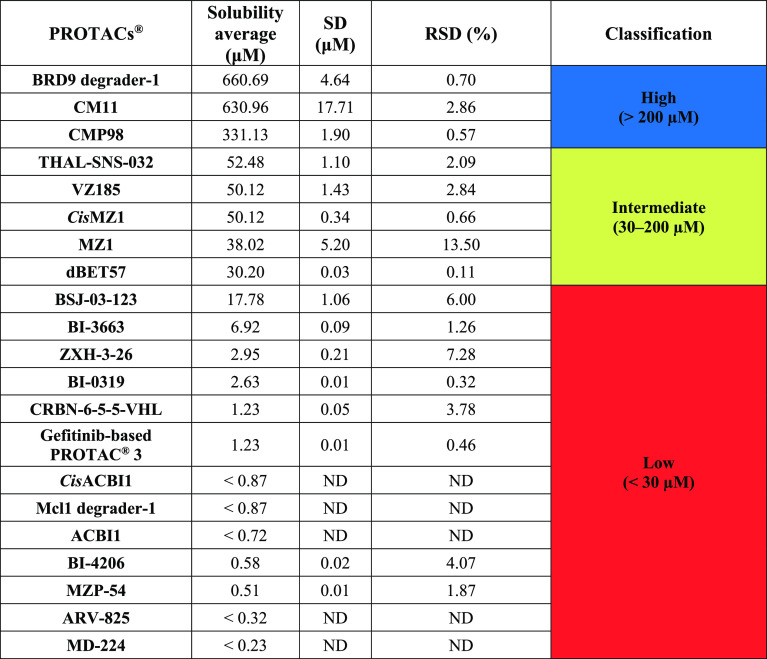
PROTAC Solubility Classification Based on GSK Guidelines^a^

a SD, standard deviations of the solubility measurements; RSD, relative standard deviations with respect to the mean; ND, not detectable

Notably, visual inspection of Figure 1 suggests that the combination of nC, PHI, and TPSA does not allow the identification of regions with different solubilities.

### Computed Solubility

Solubility can be predicted using different algorithms; nevertheless, a thorough review of them is beyond the scope of this paper (two recent reviews have been published by Abramov^25^ and Bergström and Larsson^26^). Solubility can be predicted using two approaches: quantitative structure–property relationships (QSPRs), which includes the general solubility equation (GSE), and physics-based methods based on modeling of the thermodynamic cycle. In early drug discovery there is a tendency to use simple, fast and cheap calculators implementing QSPR models. In this study we used a pool of *in silico* calculators summarized in Table S4 that implement different algorithms. Most of them (except for VolSurf) are free and are available online.

**Figure 2:**
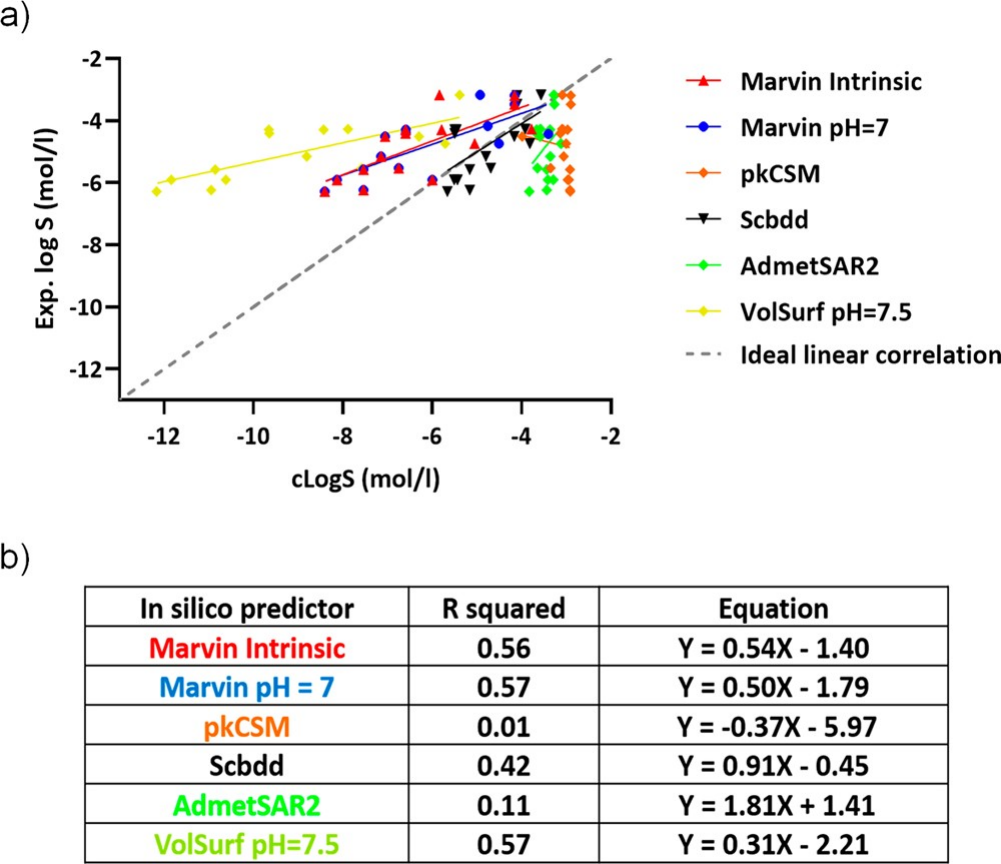
(a) Calculated versus experimental solubility for 16 PROTACs. (b) Linear regression of solubility predictors with solubility.

Experimental values for PROTACs were plotted against the predicted solubility values (Figure 2a). Moderate correlations were found (Figure 2b) between experimental log *S* and intrinsic MarvinSketch (*R*^2^ = 0.56), MarvinSketch at pH 7 (*R*^2^ = 0.57), and VolSurf (*R*^2^ = 0.57) data. Scbdd (*R*^2^ = 0.42), pkCSM (*R*^2^ = 0.01), and AdmetSAR2 (*R*^2^ = 0.11) performed worse. Notably, even though some models display moderate regressions, their slopes are considerably different from 1 (ideal linear regression). Overall, fast and cheap tools routinely used in drug discovery are not able to predict PROTAC solubility. This could be due to the lack of PROTAC experimental data in the model training sets.

### Physicochemical Descriptors Governing Solubility

#### Experimental Physicochemical Descriptors

**Figure 3:**
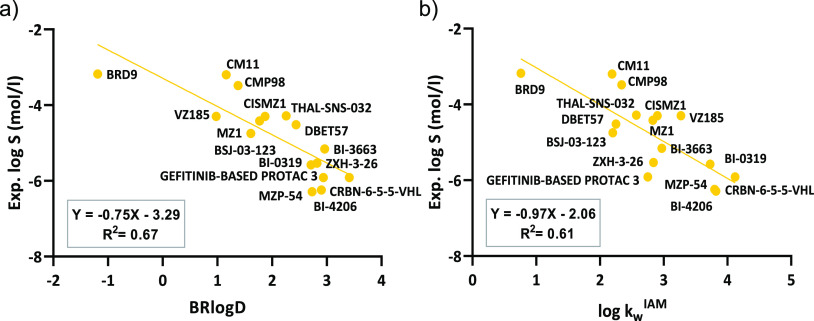
(a) Experimental solubility versus BRlogD for the PROTAC data set. (b) Experimental solubility versus log *k*_w_^IAM^ for the PROTAC data set.

PROTAC experimental solubility was first correlated with lipophilicity descriptors (log *P* is implemented in the general solubility equation (GSE)^27^). As described by some of us,^16^ BRlogD represents a validated,^28^ very useful and straightforward chromatographic descriptor for the logarithm of the distribution coefficient in the *n*-octanol/water system (log *D*) of neutral and cationic bRo5 molecules. The experimental solubilities for PROTACs were plotted against BRlogD (Figure 3a), and a promising linear correlation was found (*Y* = −0.75*X* – 3.29, *R*^2^ = 0.67, *n* = 16). As expected, the lower the BRlogD, the higher the solubility.

The logarithm of the capacity factor of an IAM column system, extrapolated at 100% water (log *k*_w_^IAM^), is also an experimental descriptor of the lipophilicity of drugs since it mimics the interaction between the polar heads of the membrane phospholipids and the drug in solution.^29^ The plot between the experimental solubility and log *k*_w_^IAM^ (Figure 3b) again reveals a consistent linear trend (*Y* = −0.97*X* – 2.06, *R*^2^ = 0.61, *n* = 16).

**Figure 4:**
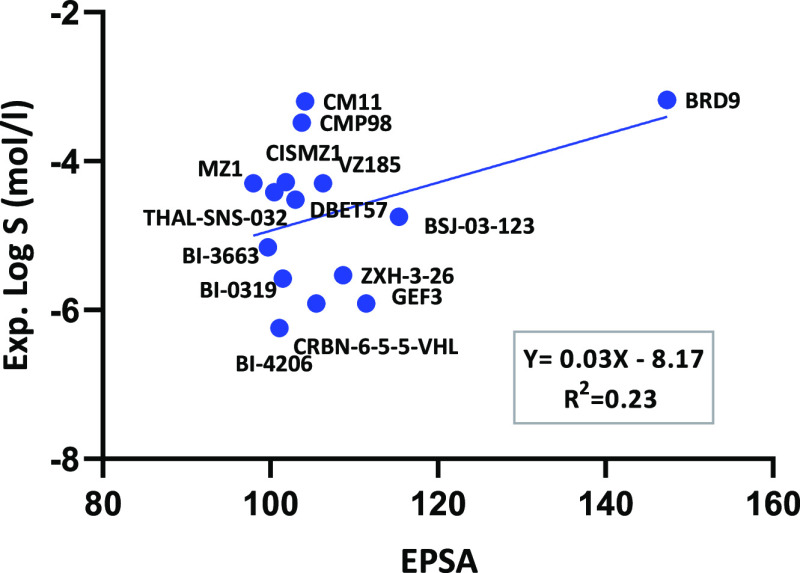
Experimental solubility versus EPSA for the PROTAC data set.

Since polarity is a molecular property often related to solubility,^13^ experimental descriptors of polarity (Δ log *k*_w_^IAM^ and EPSA) were also determined. Introduced by Grumetto in 2012,^30^ Δ log *k*_w_^IAM^ is the difference between the experimental log *k*_w_^IAM^ and the value expected for neutral analytes with a zero value of polar surface area (which depends on the experimental measurement of the *n*-octanol partition coefficient^31^). EPSA quantifies the polarity of a molecule using a supercritical fluid chromatographic (SFC) method.^32^ Notably, Δ log *k*_w_^IAM^ and EPSA (and TPSA) have been shown to provide different information about the polarities of compounds.^33^ PROTAC solubility was plotted against EPSA, but a poor positive linear correlation was found (*R*^2^ = 0.23) (Figure 4). The values of Δ log *k*_w_^IAM^ followed the same trend (*R*^2^ = 0.09) (Figure S2). Despite the poor correlation, it should be noticed that the three most soluble PROTACs (CM11, CMP98, and BRD9) have high Δlog *k*_w_^IAM^ values. Moreover, no correlation was found between the two experimental descriptors (not shown). Overall, these two experimental polarity descriptors do not seem suitable to efficiently model solubility.

#### Calculated Physicochemical Descriptors

**Figure 5:**
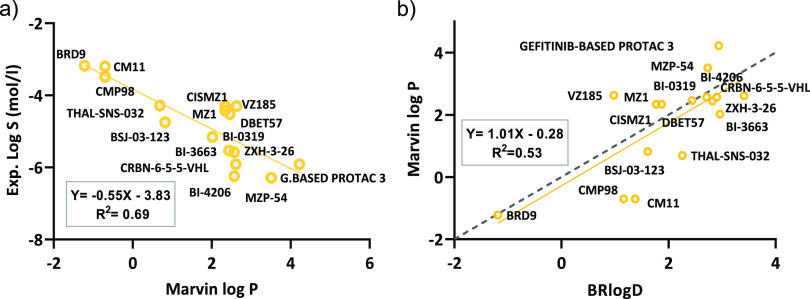
(a) Experimental solubility versus Marvin log *P* for the PROTAC data set. (b) Marvin log *P* versus BRlogD for the PROTAC data set. Ideal linear correlation is presented as a dashed line.

Although calculated log *P* values seem to be inaccurate descriptors of lipophilicity in the bRo5 chemical space,^16^ a few 2D *in silico* log *P* descriptors were used to model solubility. A representative subset of log *P* methods (atom based, fragment based, chemical descriptor based, 3D based, etc.) was selected (Table S5), and the best correlation was found for Marvin log *P* (*Y* = 0.55*X* – 3.83, *R*^2^ = 0.69, *n* = 16) (Figure 5a, Table S6). The analysis suggests that atom- and fragment-based models are more suitable calculators than Lipinski’s MLOGP. Moreover, the inclusion of a third-dimensional component (VolSurf+) did not improve the considered models. Notably, also log *D* values (when available) were not able to perform better than log *P* values. In addition, even though Marvin log *P* and BRlogD show the same correlation trends with respect to experimental solubility, they are only moderately correlated among themselves (*Y* = 1.01*X* – 0.28, *R*^2^ = 0.53, *n* = 16) (Figure 5b).

**Figure 6:**
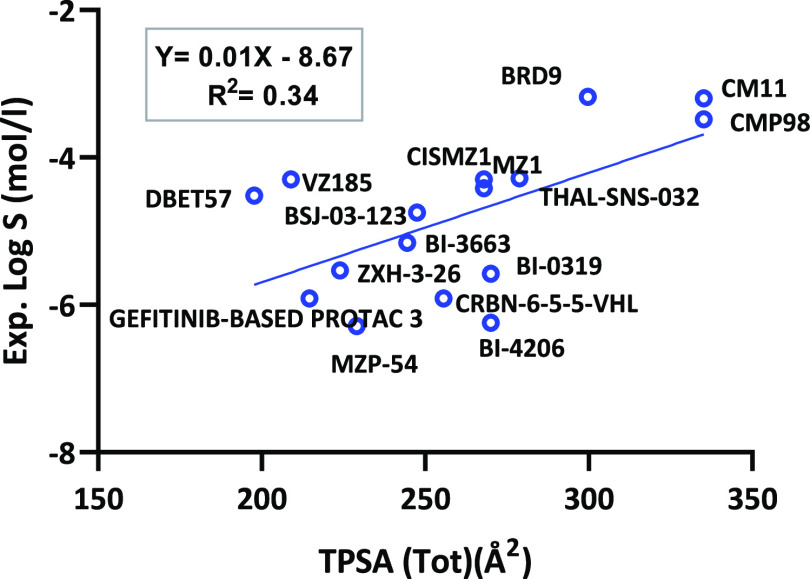
Experimental solubility versus TPSA for the PROTAC data set.

Regarding polarity, TPSA (the most common 2D computational polarity descriptor) was plotted against solubility (Figure 6). The scatter plot reveals that solubility is weakly correlated with TPSA (*Y* = 0.01*X* – 8.67, *R*^2^ = 0.34, *n* = 16). However, due to the distribution of data, if dBET57 and VZ185 were considered as outliers, *R* would rise to *R*^2^ = 0.64, revealing a moderate correlation (*n* = 14). Furthermore, TPSA was compared to Δ log *k*_w_^IAM^ and a poor correlation was found (*R*^2^ = 0.08) (Figure S3).

**Figure 7:**
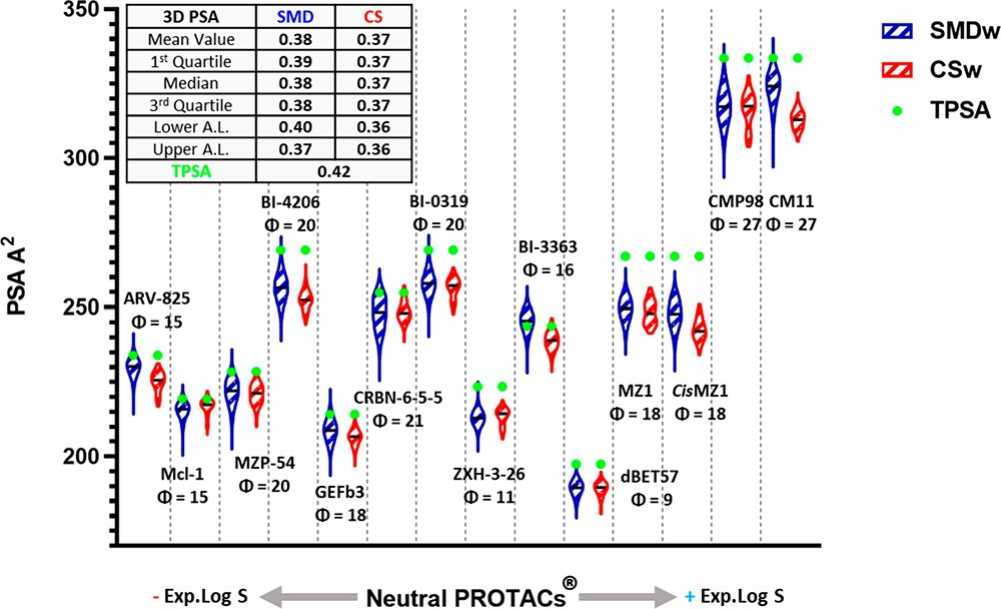
Violin plot representation of SMD (blue) versus CS (red) ordered by experimental log *S*. PHI values (flexibility) are expressed as Φ. Medians are presented as black horizontal lines. *R*^2^ are present for every statistical group (ARV-825 and Mcl-1 are not considered in the statistical analysis, since accurate solubility values are not available); the lower adjacent value (Lower A.L.) is the smallest value that is equal to or higher than the lower inner fence (first quartile – 1.5 × interquartile range). The upper adjacent value (Upper A.L.) is the highest value that is equal to or smaller than the upper inner fence value (third quartile + 1.5 × interquartile range).

Due to the poor linear correlation of experimental polarity descriptors and TPSA to experimental log *S*, we decided to explore 3D polar surface area (3D-PSA) descriptors. In fact, Kihlberg’s lab showed that for a series of bRo5 drugs the correlation between solubility and polarity improved substantially when the three-dimensional structure was taken into account.^13^ Among the plethora of available tools to generate conformers, we used the conformational sampling method implemented in the commercial Maestro package (CS) and a procedure of steered molecular dynamics (SMD) to explore a wider conformational space for 14 neutral PROTACs.^34−36^ For all the conformers arising from both CS and SMD, we calculated 3D-PSA. Then, we selected a set of representative 3D PSA values (lower adjacent limit, first quartile, median, third quartile, and upper adjacent limit) and verified their relationship with the experimental log *S*. Figure 7 suggests that, even though SMD (blue violins) extends the 3D polarity span toward the TPSA value (green dots) in comparison to CS (red violins), the *R*^2^ values do not show any improvement when replacing TPSA with 3D-PSA (*R*^2^ = 0.42). Moreover, it should be noticed that the correlation does not significantly decrease when considering different PSA regions (e.g., TPSA compared to lower adjacent limit). A rationale for this is suggested by Kihlberg’s group, who experimentally verified for PROTAC-1 the existence of water conformations displaying a wide variety of polarity and size ranges.^37^ Therefore, the fact that a single conformer (or a restricted group of conformers) does (do) not model solubility better than others, starkly supports their findings, hinting toward a solution equilibrium among conformers widely differing in polarity.

Finally, to understand the influence of other pure 2D structural descriptors on solubility data, a Bravais–Pearson correlation matrix (linear correlation) was performed (not shown). The analysis confirmed that Marvin log *P* and TPSA are the most relevant molecular properties impacting solubility. In particular, also the potential impact of size (MW) on solubility was investigated and no direct correlation was found (*R*^2^ = 0.02) (Figure S4).

Overall, computed molecular descriptors suggest that, although lipophilicity seems to play a major role in governing solubility, a contribution of polarity cannot be neglected.

### A Solubility Decision-Making Tool

**Figure 8:**
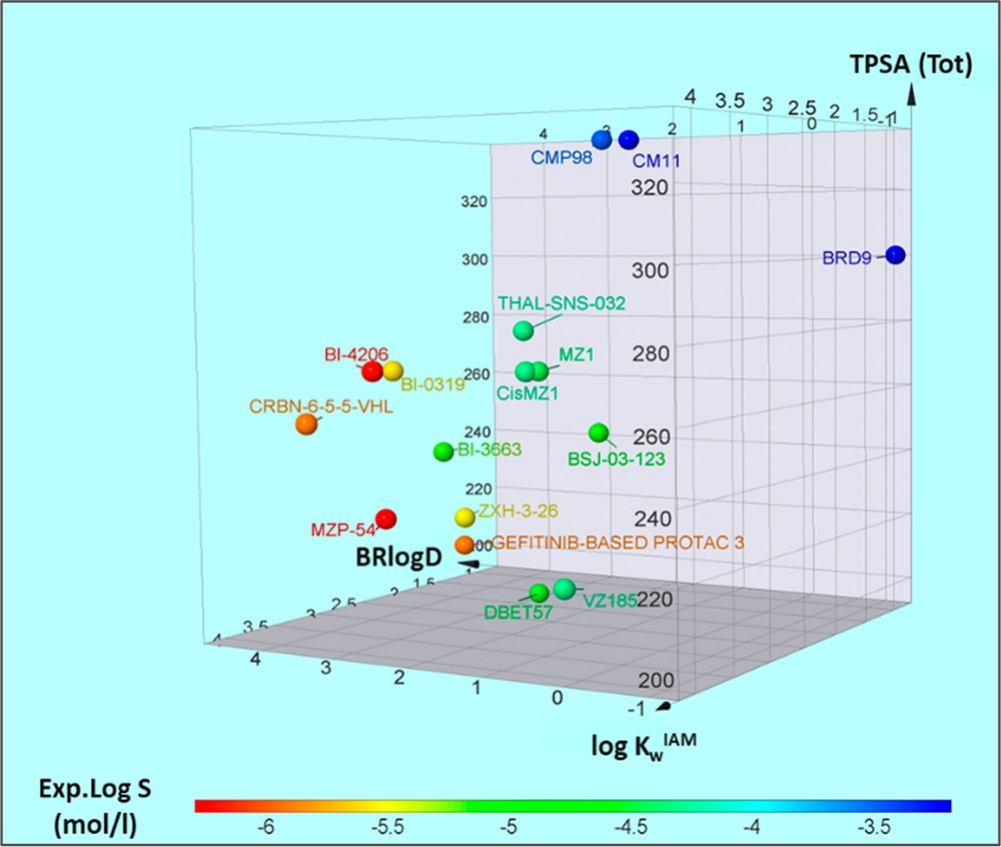
PROTAC solubility distribution based on log *k*_w_^IAM^, BRlogD, and TPSA (3D plot).

Results reported in the previous sections highlighted a significant capacity of polarity and lipophilicity in governing solubility. Therefore, the 16 PROTACs with measurable log *S* were plotted in a 3D scatter plot based on log *k*_w_^IAM^, BRlogD, and TPSA values (Figure 8). Moreover, PROTACs were also colored according to the three solubility groups introduced by GSK in Table 1 (Figure S5). Notably, in Figure S5 only BSJ-03-123 was graphically misclassified.

In practice Figure 8 shows that thermodynamic solubility could be efficiently classified using two chromatographic descriptors (log *k*_w_^IAM^, BRlogD) and one computational descriptor (TPSA). This finding represents a promising tool for drug discovery since the experimental determination of the two chromatographic descriptors is easily automated and less time-consuming than standard solubility protocols.

**Figure 9:**
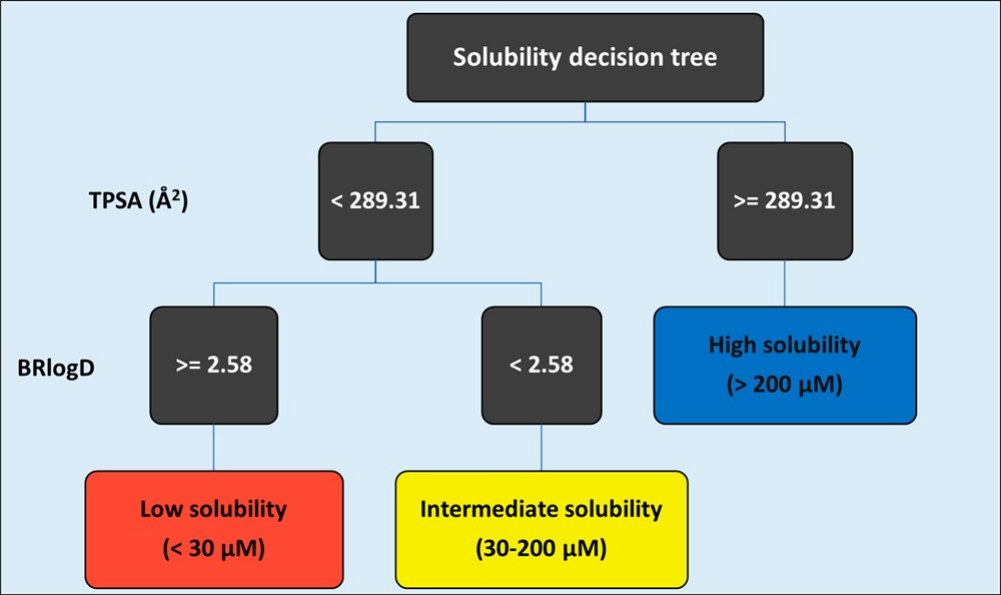
PROTAC random tree model (BRlogD and TPSA) colored by the experimental classification: low (red), intermediate (yellow), and high (blue).

The promising PROTAC solubility distribution (Figure 8) makes a claim for setting up a solubility classification model. We are aware that the low number of data could bias the results, but we were interested in verifying whether machine learning models are coherent with the graphical output reported in Figure 8. Therefore, the solubility matrix was added with the three GSK classes (low, intermediate, and high solubility; Figure S5) and submitted to random forest and decision random tree algorithms implemented in Weka.^38^ Random tree adopts a supervised and fast algorithm that makes a prediction guided by the outcome, suffering from overfitting. Random forest, on the other hand, performs multiple random decisions, obtaining an outcome directed exclusively by the majority of the results. Since Figure S5 suggested that BSJ-03-123 was misclassified in the low solubility group, we removed it from the models (*n* = 15 instances). The resulting models (verified by 10-fold cross-validation) revealed that 86.7% of the instances were correctly classified for both models (Figure S6). Therefore, it seems that the chosen algorithm does not influence the output of this preliminary study. Moreover, the confusion matrices (Figure S6) suggested that low solubility PROTACs were correctly predicted (100%), whereas intermediate and high solubility PROTACs were worse predicted (80 and 66.7%, respectively). Consequently, the random tree algorithm provided a definitive model based on BRlogD and TPSA (Figure 9): a TPSA value equal to or higher than 289.31 Å^2^ directly classifies PROTACs as highly soluble, whereas less polar PROTACs having BRlogD values equal to or higher than 2.58 are classified into the low solubility group. These results confirm the hypothesis suggested by the 3D plot: high polarity and low lipophilicity favor solubility.

Notably, even though the three descriptors were considered, the models only took into account BRlogD and TPSA for the decision-making, probably because a polarity descriptor provides more complementary information over a second lipophilicity descriptor.

Overall, for a small data set of not structurally related PROTACs, the combined use of the 3D graph and the random tree model seems a reasonable strategy to classify solubility. This result could be assumed as a starting point to set up efficient tools to be implemented in pharma companies drug discovery pipelines.

### Impact of Building Blocks on PROTACs Solubility

In the previous sections we showed how molecular properties such as lipophilicity and polarity may help to rationalize the different solubility properties exhibited by the investigated data set of PROTACs. However, since PROTACs are synthesized by combining either three (warhead, linker, and E3 ligand) or two (E3 ligand bound to the linker and warhead) molecules, medicinal chemists are also interested in knowing the contribution of building blocks to the whole PROTAC solubility. In practice, it would be crucial for instance to know whether the solubility difference of two PROTACs sharing the same E3 ligand and the same linker could be predicted from the solubility of their warheads (Table S1). Since we are dealing with large and flexible molecules affected by intramolecular interactions, *a priori* we cannot be sure that a given molecular property (e.g., solubility) can be computed by summing up the contributions of the three moieties.

To shed light on this aspect, in the data set of the 21 PROTACs we identified three pairs differing in the warhead, linker, and E3 ligase ligand, respectively. Then we retrieved reasonable building blocks commercially available, determined their experimental and predicted solubilities (Table S7), and found the previously discussed molecular descriptors (Table S8). Finally, we discussed the solubility difference between pairs based on the building block properties.

**Figure 10:**
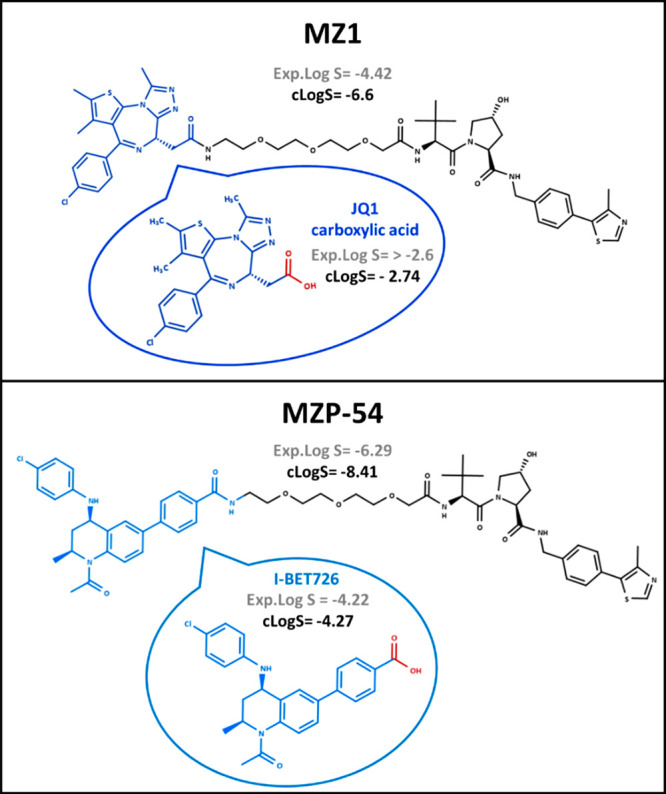
Comparison between MZ1 and MZP-54. Experimental (Exp.Log S, *S* in mol/L) and/or calculated (cLogS, Marvin pH 7) values are presented for PROTACs and their warheads.

To investigate the warhead contribution to solubility, we used the pair MZ1–MZP-54 (Figure 10). MZ1^39^ is a selective degrader of BRD4 over BRD2 and BRD3, whereas MZP-54^40^ selectively degrades BRD3 and BRD4 over BRD2. Structurally, MZ1 and MZP-54 (Figure 10) share the same E3 ligand and linker, differing exclusively in the warhead (JQ1 and I-BET726, respectively).

Both PROTACs are neutral at pH 7 as verified by Caron’s group.^41^ Our experimental solubility data (*S* in moles per liter) support that MZ1 (log *S* = −4.42) is significantly more soluble than MZP-54 (log *S* = −6.29). In this example, both experimental and computed solubilities of the warheads (blue in Figure 10) justify the solubility difference of the two PROTACs. In fact, JQ1 carboxylic acid (warhead of MZ1) is significantly more soluble (log *S* > −2.6) than I-BET726 (warhead of MZP-54, log *S* = −4.42). As expected, JQ1 carboxylic acid displays lower BRlogD and log *k*_w_^IAM^ values and higher TPSA values with respect to I-BET726 (Table S8). Therefore, this pair suggests that classification of PROTAC solubility from its building blocks is feasible.

**Figure 11:**
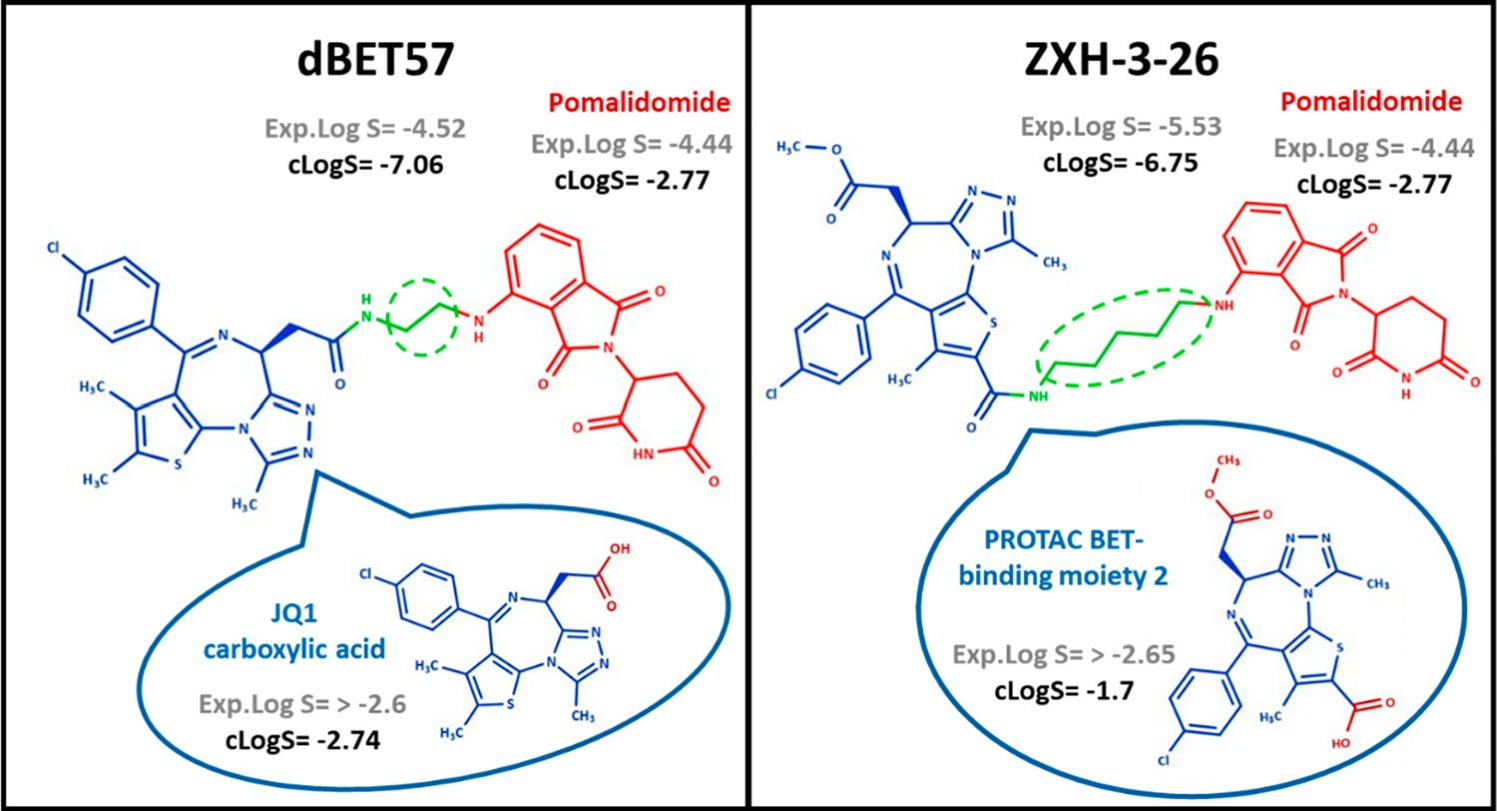
Comparison between dBET57 and ZXH-3-26. Experimental and/or calculated log *S* (*S* in mol/L) values (Marvin pH 7) are presented for PROTACs and their building blocks.

To explore the impact of the linker on the PROTAC solubility, we used the pair dBET57–ZXH-3-26 (Figure 11). dBET57^42^ and ZXH-3-26^42^ are selective BRD4 degraders that share the same E3 ligase (pomalidomide) and a similar warhead (JQ1 in dBET57 and PROTAC BET-binding moiety 2 in MZP-54), differing largely in the linker length (green dashed circle in Figure 11).

Both PROTACs are expected to be neutral at pH 7. Experimental solubility shows that dBET57 (log *S* = −4.52) is more soluble than ZXH-3-26 (log *S* = −5.53) by about 1 log unit. Notably, Marvin pH 7 calculated values suggest otherwise (−7.06 vs −6.75).

The warhead named PROTAC BET-binding moiety 2 (shown in blue in Figure 11) shows an additional methyl ester moiety (colored in dark red) when compared to JQ1 carboxylic acid. Both warheads show optimal experimental solubilities despite their structural differences. However, we cannot say if one is more soluble than the other. Therefore, in the first approximation, the considerable solubility difference of this PROTAC pair is expected to be due to the linker contribution. In this respect, dBET57 incorporates a more soluble alkyl linker (ethylamine) than ZXH-3-26 (pentyl-1-amine). Therefore, the different linker length could justify a decrease of about 1 experimental log *S* unit. However, it should be observed that the two warheads bind the linker in a different position and that the extra ester group of ZXH-3-26 may impact solubility. Esters are expected to improve solubility, but this could not happen if the ester group was involved in the formation of an intramolecular hydrogen bond (IMHB) which in turn could be facilitated by the higher flexibility of the compound due to the presence of a longer linker. Overall, this example supports that the different solubilities of the two PROTACs cannot easily be related to the different chemical structures of the linker.

**Figure 12:**
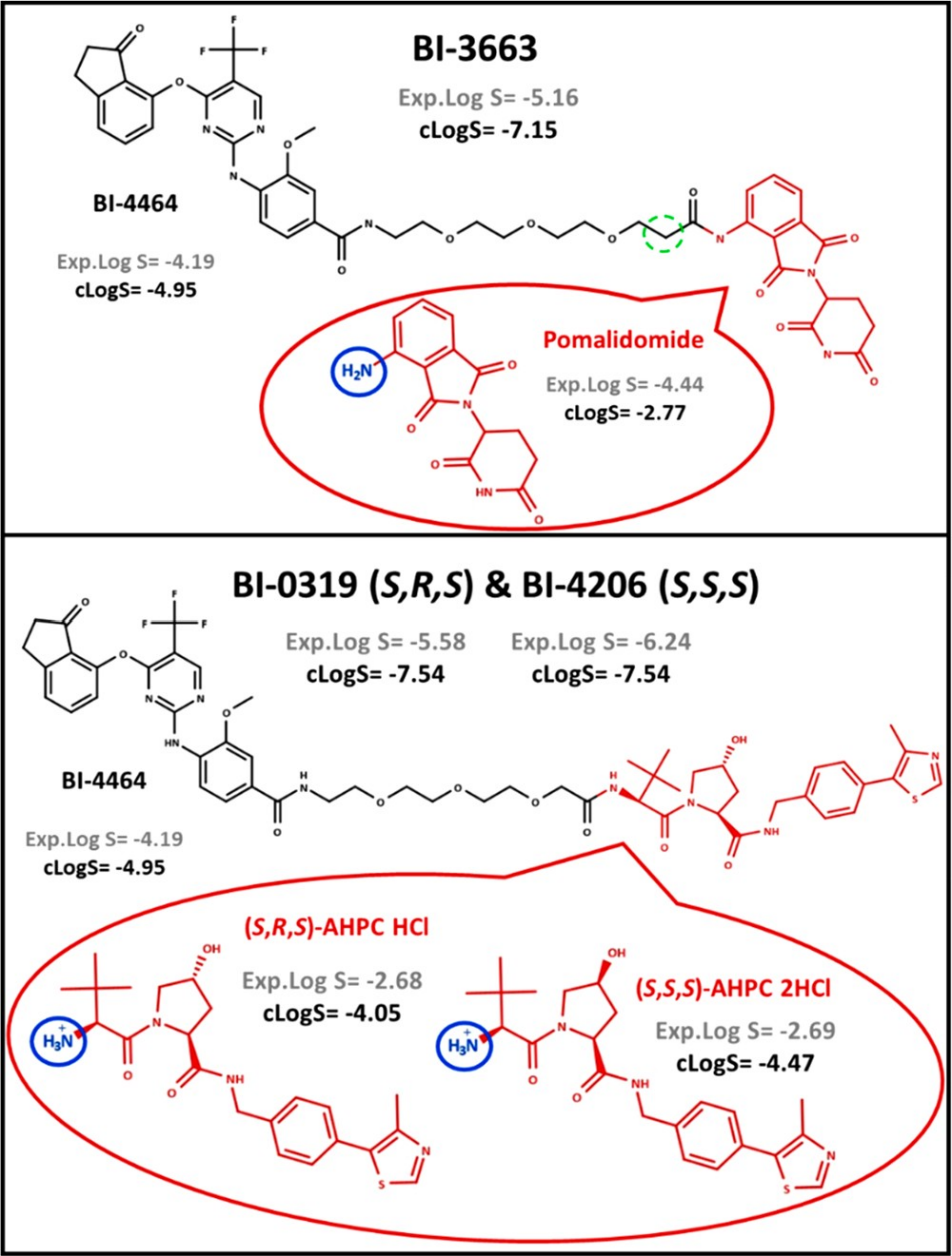
Comparison between BI-3663 and BI-0319/BI-4206. Experimental and/or calculated log *S* (*S* in mol/L) values (Marvin pH 7) are presented for PROTACs and their building blocks.

To investigate the E3 ligase ligand contribution to solubility, we used the PROTAC pair BI-3663–BI-0319/BI-4206^43^ (Figure 12). BI-3663 and BI-0319 degrade the PTK2 protein, and BI-4206 is the negative control for BI-0319. They all share the same warhead (BI-4464), show a similar long linker bearing one extra carbon for BI-3663 (dashed circle in green), and differ in the E3 ligase ligand (pomalidomide and VH032, respectively).

Both calculated and experimental solubility values support that BI-3663 is more soluble (log *S* = −5.16) than BI-0319/BI-4206 (log *S* = −5.58 and −6.24, respectively) (Figure 12). In addition, the higher solubility expressed by BI-3663 is in line with the higher computed solubility of pomalidomide in comparison with VHL derivatives. However, experimental determinations revealed that pomalidomide is less soluble (−4.44) than *S*,*R*,*S*-AHPC HCl and *S*,*S*,*S*-AHPC 2HCl (−2.68 and −2.69, respectively). The reason for this contradictory behavior probably relies on the different ionization profiles of the investigated building blocks and their variation when included in the PROTAC structure. The two VHL diastereoisomers are protonated at pH 7 (in blue in Figure 12), as determined by potentiometry (Table S8). Conversely, pomalidomide is not ionized at pH 7. Furthermore, a recent study focusing on cereblon ligands has proved that the stabilized π-electron system and the capacity of the aromatic nitrogen to form an intramolecular hydrogen bond with the neighbor oxygen atom can increase lipophilicity, with a notable worsening of pomalidomide’s water solubility.^44^ Therefore, since solubility was measured at pH 7, the charged state of the two VHLs justifies their higher experimental solubilities over pomalidomide. However, the ionization center is lost in BI-0319/BI-4206 because of the formation of an amide group. Conversely, pomalidomide remains neutral when considered as part of BI-3663’s structure. Consequently, even though *S*,*R*,*S*-AHPC HCl and *S*,*S*,*S*-AHPC 2HCl are more soluble than pomalidomide when considered as independent structures, they trigger a less soluble PROTAC.

Overall, these arguments highlight the issues in predicting solubility from building blocks when they do not share the same terminal groups.

## Conclusions

Solubility is a crucial molecular property strongly impacting the future of PROTACs as oral drugs. Its determination, prediction, and understanding should be faced starting from early drug discovery. This process should also include considerations about permeability since the solubility–permeability interplay must be taken into account to obtain the optimal solubility–permeability balance, in order to maximize the overall absorption.

Overall, in this study we combined experimental and computational strategies to explore the solubility behavior of 21 commercial PROTACs also in relation to their building blocks. Therefore, we provide a data set of solubility, lipophilicity, and polarity data experimentally determined with previously validated methods. Then we proved that at least up to now these data cannot be predicted by common calculators largely employed in Ro5 drug discovery pipelines. The interplay between solubility, lipophilicity, and polarity was confirmed also for PROTACs. The role of the third dimension in modulating solubility seems to be modest as revealed by conformational sampling and steered molecular dynamics. Very interestingly, we also verified that deducing the solubility of PROTACs from the solubility of building blocks is generally risky, although feasible under some circumstances.

Taken together, these results allow us to set up an automated and straightforward strategy to classify PROTACs in their solubility based on one polarity (computed) and two lipophilicity (chromatographic) descriptors. Moreover, with the help of machine learning, we propose BRlogD and TPSA as key indicators of PROTAC solubility, where 2.58 and 289 Å^2^ are their respective thresholds for an experimental solubility classification. Nevertheless, it should not be forgotten that PROTACs displaying moderate or poor predicted solubility may benefit from pharmaceutical formulations. Obviously, this study does not pretend to exhaustively deconvolute the complexity of solubility in the PROTAC chemical space, but it fixes some guidelines that are expected to improve the efficiency and speed of the next PROTAC-based early drug discovery campaigns.

## Experimental Section

### Data Set Selection

A series of 21 PROTACs and 7 building blocks were selected and bought based on their commercial availability, or they were freely delivered in the pipeline of the collaborative pharmaceutical companies. All the investigated compounds are >95% pure by HPLC analysis. Moreover, the selection of building blocks was based on the structural similarity to the theoretical building block contributing to the full PROTAC structure.

### Materials

The series of PROTACs and building blocks were bought or supplied by different pharmaceutical companies (Tables S9 and S10, respectively). All other chemical reagents were of analytical grade. HPLC grade acetonitrile (ACN) and methanol were bought from VWR Chemicals. Ammonium acetate was provided by Alfa Aesar, and KCl was from Sigma-Aldrich. DMSO was purchased from Sigma-Aldrich, quality EMSURE ACS. Potassium phosphate monobasic (KH_2_PO_4_) and dipotassium phosphate (K_2_HPO_4_) were provided by Carlo Erba Reagents, ACS grade. MS grade ammonium formate was bought from Merck. Carbon dioxide 4.5 grade was purchased from SOL Group. A syringe filter (4 mm) and 0.45 μm PTFE membrane from GE Healthcare Life Sciences and Milli-Q water were used.

### Instruments

Solids were weighed with a Sartorius Entris224-1S Analytical (www.sartorius.com). p*K*_a_ values were measured with a SiriusT3 instrument (Sirius Analytical Instruments) equipped with a reference pH electrode (Ag/AgCl double junction) and a turbidity detector. Ultrasonic cleaner, obtained from VWR Chemicals (www.vwr.com), and an IKA VORTEX 3 (www.ika.com) were used to avoid precipitation in the calibration points. Stirring and heating were provided by magnetic and heating plates C-MAG MS 7 and IKA ETS-D5, respectively (www.ika.com). Moreover, pH was measured with a Eutech pH Meter 2700 (www.fishersci.com). Analyses were carried out by high-performance liquid chromatography (HPLC; DIONEX Ultimate 3000, Thermo Scientific Inc.) provided with an RS diode array and Chromeleon 7.2.10 software (www.thermofisher.com). HPLC columns IAM.PC.DD2 (300 Å, 10 μm, 10 cm × 4.6 mm) from REGIS and XBridge Shield RP18 (130 Å, 5 μm, 5 cm × 4.6 mm) from Waters (www.waters.com) were used. Ergonomic high-performance single-channel variable volume pipettors, HPLC 1.5 mL vials, 0.1 mL microinsert, and PP 9 mm screw caps were obtained from VWR Signature. EPSA analyses were performed by supercritical fluid chromatography (SFC; JASCO SFC-4000, Jasco Europe srl), provided with a diode array and ChromNAV 2.04.00 software (www.jascoweb.com). The SFC column Chirex 3014 (*S*)-VAL and (*R*)-NEA (100 Å, 5 μm, 25 cm × 4.6 mm) from Phenomenex was used.

### p*K*_a_ Determination

Acidic dissociation constants of pomalidomide, *S*,*R*,*S*-AHPC HCl, and *S*,*S*,*S*-AHPC 2HCl were measured potentiometrically. Titrations were performed to 0.15 M KCl sample solutions under a nitrogen atmosphere at 25 ± 1 °C. Moreover, standardized 0.5 M KOH and 0.5 M HCl were used as titration reagents (solutions were prepared from Titrisol (Merck) concentrated solutions).^45^

### Solubility Determination

#### Sample Preparation

Solubility was determined in 10 mM PBS (0.15 M KCl) at pH 7, 25 °C. A 1–2 mg sample of each molecule was added to the same quantity (1–2 mL) of the mentioned buffer to obtain 1 mg/mL solutions in the vial. Samples were then submitted to 25 °C and magnetic stirring (500 rpm) for 1 h. After this period, solutions or suspensions were filtered through the 0.45 μm membrane pore and diluted with buffer, 10 mM PBS (0.15 M KCl). The amount of dissolved compound in each sample was then quantified, in duplicate, via HPLC using ultraviolet (UV) spectrometric detection.

#### Calibration Curves

Variable volumes of pure DMSO (0–100 μL) were added separately to small and defined (1 mg) quantities of each compound to obtain clear solutions. Solutions were then adjusted with 10 mM PBS (0.15 M KCl) to reach 5% DMSO 10 mM PBS (0.15 M KCl), resulting in particle precipitation in most of the cases. Afterward, 5% DMSO 10 mM PBS (0.15 M KCl) was added until the suspensions or precipitations were redissolved, to render clear solutions. As soon as solutions were obtained, they were used as mother solutions for the performance of 5–10 point calibration curves with serial buffer dilutions (1:1; v/v) 5% DMSO 10 mM PBS (0.15 M KCl). Calibration points were then measured via HPLC-UV. Each compound was soluble at a different concentration, and consequently, each compound required its own calibration curve.

#### HPLC Methods

The mobile phase consisted of a solution of acetonitrile (ACN) and 20 mM ammonium acetate buffer (AAB), pH 7, freshly prepared. A 10 μL volume of each sample (volume of injection) was injected at an isocratic 1 mL/min flow rate analyzed at 30 °C (oven temperature). Solubility determination of each compound required a specific HPLC method. Settings are collected in Table S11 (PROTACs) and Table S10 (building blocks).

#### Quantification

Injections were performed in duplicate for each molecule’s calibration points. The retention time and area under the curve (AUC in absorbance units) were collected, and the average was calculated. The AUC average (*y*-axis) was plotted versus theoretical concentration (mg/mL; *x*-axis) to obtain a calibration curve for each molecule. Correlation equations and coefficients (*R*^2^) were efficiently calculated for each compound (Table S12 for PROTACs and Table S10 for building blocks). Samples were then injected in duplicate, and the AUC averages and standard deviations were calculated. Finally, the absorbance averages were interpolated into the corresponding equations, extracting the final solubility values with their standard deviation, after dilution factor corrections.

### BRlogD

The mobile phase consisted of an isocratic solution of 20 mM ammonium acetate (pH 7.0) and acetonitrile, 40–60%, respectively (v/v).^16^ Samples were dissolved in buffer/ACN and injected into the XBridge column at a flow rate of 1.0 mL/min at 30 °C. Retention times were determined in duplicate, and the dead time, *t*_0_, was recorded as the baseline disturbance. Consequently, log *k*′_60_ was calculated (capacity factor *k*′_60_ = [*t*_R(60% ACN)_ – *t*_0_]/*t*_0_) and transformed into the corresponding BRlogD value with the equation BRlogD = 3.31*x* + 2.79.^16^

### log *k*_w_^IAM^ (Lipophilicity) and Δ log *k*_w_^IAM^ (Polarity) Determination Using IAM Systems^17^

#### log *k*_w_^IAM^

The mobile phase consisted of a solution of 20 mM ammonium acetate (pH 7.0) in a mixture with acetonitrile at various percentages (from 10 to 50%, v/v). Samples were dissolved in buffer/ACN and injected into the previously mentioned IAM column at a flow rate of 1.0 mL/min at 30 °C. Chromatographic retention data were determined in duplicate for the different mobile phase conditions. Data were recorded as log *k*′ (capacity factor *k*′ = [*t*_R_ – *t*_0_]/*t*_0_), where *t*_R_ and *t*_0_ are the retention times of the sample and a nonretained molecule (citric acid), respectively. Thus, log *k*_w_^IAM^ values were calculated by an extrapolation method of the experimental equation, at 0% acetonitrile. Moreover, five gold standard compounds (caffeine, carbamazepine, ketoprofen, theobromine, and toluene) were checked daily.

#### Δ log *k*_w_^IAM^

It was calculated with the equation Δ log *k*_w_^IAM^ = log *k*_w_^IAM^ – clog *k*_w_^IAM^, where clog *k*_w_^IAM^ (defined as log *k*_w_^IAM^ for neutral compounds^30^ that have PSA = 0) has been correlated to BRlogD with the equation clog *k*_w_^IAM^ = BRlogD·0.92 – 1.03.^17^

### EPSA (Polarity) Determination

EPSAs were determined for the 16 PROTACs with accurate values (Table 1) following the SFC protocol by Goetz and co-workers.^32^ Briefly, a polar stationary phase (Chirex 3014) and a nonpolar mobile phase (supercritical CO_2_ with the addition of 20 mM ammonium formate in methanol as a modifier) were used to enable separation of compounds on the basis of their polarity. The modifier was varied in 11 min from 5 to 60% at 5%/min in a linear gradient, holding at 60% for 4.9 min and reverting to the original 5% in 0.1 min. The flow rate was 5 mL/min with the outlet back pressure set to 100 bar, instead of 140 bar of the original method. Samples were dissolved in DMSO, and the injection volume was 5 μL. The column temperature was set to 40 °C. Each sample was analyzed in duplicate.

### Computational Part

#### Molecular Descriptors

The SMILES codes of the PROTACs and building blocks were submitted to solubility calculators (Table S4) using 2D descriptor-based solubility models: AdmetSAR2 (www.lmmd.ecust.edu.cn/admetsar2/), ADMETLab (www.scbdd.com), and pkCSM (biosig.unimelb.edu.au/pkcsm). In addition, a 3D descriptor-based model, VolSurf+ (VS+, www.moldiscovery.com, ver. 1.1.2, 2016), and a fragment-based model, Marvin Sketch (ChemAxon, https://www.chemaxon.com, ver. 20.18.0, 2020), were also used. Log *P* was calculated with different tools (Table S5): Swissadme (www.swissadme.ch/index.php), ACD Laboratories (www.acdlabs.com), Molinspiration (www.molinspiration.com), ADMETLab (www.admet.scbdd.com), AdmetSAR2 (www.lmmd.ecust.edu.cn/admetsar2/), and pkCSM (biosig.unimelb.edu.au/pkcsm). In addition, VolSurf+, MoKa (www.moldiscovery.com, ver. 3.2.2, 2019), and Marvin Sketch and were also used.

Moreover, Kode srl, Dragon (software for molecular descriptor calculation, https://chm.kode-solutions.net/pf/dragon-7-0/, ver. 7.0.10, 2017), and AlvaDesc (Alvascience, Software for Molecular Descriptors Calculation, www.alvascience.com/alvadesc/, ver. 1.0.18, 2020) were used to calculate 2D physicochemical descriptors including nC (number of carbon atoms), PHI (Kier’s flexibility index), and TPSA. Finally, the relationship between solubility and molecular descriptors was investigated with OSIRIS DataWarrior (www.openmolecules.org/datawarrior/, ver. 5.2.1, 2021) and GraphPad Prism (www.graphpad.com, ver. 8.0.0, 2019).

#### Conformational Studies

The conformational profile was studied for a subset of 14 neutral PROTACs, using conformational sampling (CS) and steered molecular dynamics (SMD) tools.

The default conformational sampling (CS) tool implemented in the Schrödinger molecular modeling package (CS) was employed to generate relevant 3D conformations in water (Schrödinger Release 2021–3, Maestro, ver. 12.3, and Schrödinger Release 2021–3, Macromodel). For this purpose, the force field OPLS_2005 (default parameters) was applied to every 3D PROTAC structure (optimized 3D structures were previously obtained from www.mn-am.com/online_demos/corina_demo).

The SMD was set up by using the online input generator CHARMM-GUI (www.charmm-gui.org/).^46^ Each PROTAC structure (starting from an optimized geometry obtained with Corina Demo) was first converted from mol2 to a PDB file, and the relative CHARMM36 parameters were generated with the “Ligand reader and modeler” functionality of CHARMM-GUI. Then, the periodic boundaries, the structure water solvation, and the classical MD input files for an *NPT* ensemble (constant number of particles, pressure, and temperature) at 300 K were generated through the “solution builder” functionality of CHARMM-GUI. The input and parameter files were then downloaded and the production default input file was modified, introducing the code for the additional SMD velocities (parameters: SMD = on, SMDk = 7.0 kcal/mol/Å, SMDvel = 2 × 10^–5^ Å/ts, SMDdir = 0.0 1.0 0.0). The SMD atoms subset (the PROTAC structure) was defined by a modification of the occupancy field (from 0 to 1) in a PDB file recording the coordinates after system equilibration. NAMD^47^ 2.13 CUDA-accelerated version (www.ks.uiuc.edu/Research/namd/) was used to equilibrate the system (250 ps) and to run the SMD production (10 ns) on a Linux workstation (OS CentOS7, 32GB DDR2; CPU Xeon Octa-core 3.50 GHz, Titan XP GPU). Lastly, the resulting production trajectories were visualized and cleaned (eliminating the explicit solvent) with VMD^48^ (http://www.ks.uiuc.edu/Research/vmd).

Finally, the generated trajectories by CS and SMD were loaded into VEGA ZZ software (http://www.vegazz.net/)^49^ for 3D PSA calculation (probe radius 0 Å).

#### Machine Learning Classification

The Weka software^50^ (ver. 3.8.5) was used to perform a classification of PROTAC solubility based on log *k*_w_^IAM^, BRlogD, and TPSA values. To do so, supervised random tree and random forest algorithms were used (default parameters) to create efficient prediction models.

## Supporting Information Available

List of studied PROTACs and building blocks; ionization of selected PROTACs; experimental solubility values; *in silico* log *S* and log *P* predictors; correlation of computed log *P* with solubility; experimental and calculated log *S* values of PROTACs and building blocks; experimental descriptors and TPSA for selected building blocks; list of studied PROTACs, PROTAC-DB IDs, and vendor; experimental conditions for building blocks characterization; experimental chromatographic conditions for determination of PROTAC solubility; calibration curves for PROTAC data set; structures of the 21 investigated PROTACs; experimental solubility versus Δ log *k*_w_^IAM^; Δ log *k*_w_^IAM^ versus TPSA; experimental solubility versus MW; solubility distribution in chemical space; solubility classification models; HPLC traces (PDF)
Formula strings from SMILES (CSV)

## Abbreviations Used

BR: block relevance
bRo5: beyond the rule of 5
CS: conformational sampling
DMPK: drug metabolism and pharmacokinetics
IAM: immobilized artificial membranes
IMHB: intramolecular hydrogen bond
POI: protein of interest
PROTAC: proteolysis targeting chimera
SMD: steered molecular dynamics
TPD: targeted protein degradation
TPSA: topological polar surface area
