# Supplementary material for: Designing Soluble PROTACs: Strategies and Preliminary Guidelines
Source: J Med Chem. 2022 Apr 25;65(19):12639–49. doi: 10.1021/acs.jmedchem.2c00201 (PMC9574862; doi:10.1021/acs.jmedchem.2c00201)
Supplement: Supplementary file 1 — jm2c00201_si_001.pdf [file jm2c00201_si_001.pdf]

Supporting Information

Designing soluble PROTACs®: strategies and preliminary guidelines

Diego García Jiménez,[†] Matteo Rossi Sebastiano,[†] Maura Vallaro,[†] Valentina Mileo[‡,#], Daniela Pizzirani[‡,#], Elisa Moretti[‡], Giuseppe Ermondi,[†] and Giulia Caron[,†]*

[†]University of Torino, Molecular Biotechnology and Health Sciences Dept., CASSMedChem, Via Quarello 15, 10135 Torino, Italy.

[‡]Chiesi Farmaceutici, Global Research and Preclinical Development, Research Center, Largo Belloli 11/a, 43122 Parma, Italy.

[#]Chiesi Farmaceutici, Emerging Science & Technology Unit, Research Center, Largo Belloli 11/a, 43122 Parma, Italy.

Corresponding Author's email address: giulia.caron@unito.it

List of contents

Supplementary File: *Supporting information for publication.csv*, contains formula strings

Supplementary Tables:

Supplementary Table 1: list of studied PROTACs® and building blocks

Supplementary Table 2: pK_a and ionization states

Supplementary Table 3: experimental solubility values

Supplementary Table 4: *in silico* log S predictors used

Supplementary Table 5: *in silico* log P predictors used

Supplementary Table 6: correlation of computed log P with solubility

Supplementary Table 7: experimental and calculated log S of PROTACs® and selected building blocks

Supplementary Table 8: experimental descriptors and calculated TPSA for selected PROTAC® building blocks

Supplementary Table 9: list of studied PROTACs®, PROTAC-DB IDs and vendor

Supplementary Table 10: experimental conditions for the characterization of the building blocks

Supplementary Table 11: experimental chromatographic conditions for the determination of PROTAC® solubility.

Supplementary Table 12: calibration curves for the PROTAC® dataset

Supplementary Figures:

Supplementary Figure 1: structures of the 21 investigated PROTACs®

Supplementary Figure 2: experimental solubility vs $\Delta \log k_w^{IAM}$ for the PROTAC® dataset

Supplementary Figure 3: $\Delta \log k_w^{IAM}$ vs TPSA for the PROTAC® dataset

Supplementary Figure 4: Experimental solubility vs MW for the PROTAC® dataset.

Supplementary Figure 5: PROTAC® solubility distribution in the chemical space

Supplementary Figure 6: Solubility classification models

Supplementary Figure 7: HPLC traces

Table S-1: List of the studied PROTACs and their predicted constitutive building blocks (information obtained directly from the PROTAC-DB: http://cadd.zju.edu.cn/protacdb/). Building blocks are presented as the fundamental ideal constituents of the PROTAC, regardless of the real building blocks structures used for the synthetic approach.

PROTACs	DB-ID	Warhead	Linker	E3 ligand
**ACBI1**	798	Undefined	1-ethoxy-4-methylbenzene	VH101: (S,R,S) VH032-cyclopropane-F
**ARV-825**	329	Birabresib (OTX-015)	1-ethoxy-2-(2-ethoxyethoxy)ethane	Pomalidomide
**BI-0319**	1656	BI-4464	1-(2-ethoxyethoxy)-2-methoxyethane	VH032: (S,R,S)-AHPC
**BI-3663**	1655	BI-4464	3-[2-(2-ethoxyethoxy)ethoxy]propanal	Pomalidomide
**BI-4206**		BI-4464	1-(2-ethoxyethoxy)-2-methoxyethane	VH032, negative control: (S,S,S)-AHPC
**BRD9 degrader-1**	1187	GSK-39	N-(4-acetamidobutyl)-2-hydroxyacetamide	Thalidomide
**BSJ-03-123**	240	Palbociclib	N-[2-[2-(2-ethoxyethoxy)ethoxy]ethyl]-2-hydroxyacetamide	Thalidomide
***Cis*ACBI1**		Undefined	1-ethoxy-4-methylbenzene	VH101, negative control :(S,S,S) VH032-cyclopropane-F
***Cis*MZ1**		JQ1	2-[2-(2-methoxyethoxy)ethoxy]ethanamine	VH032, negative control: (S,S,S)-AHPC
**CM11**	998	VH032: (S,R,S)-AHPC	1-methoxy-2-[2-[2-[2-(2-methoxyethoxy)ethoxy]ethoxy]ethoxy]ethane	VH032: (S,R,S)-AHPC
**CMP98**		VH032, negative control: (S,S,S)-AHPC	1-methoxy-2-[2-[2-[2-(2-methoxyethoxy)ethoxy]ethoxy]ethoxy]ethane	VH032, negative control: (S,S,S)-AHPC
**CRBN-6-5-5-VHL**	176	Pomalidomide	1-(5-butoxypentoxy)hexane	VH032: (S,R,S)-AHPC
**dBET57**	342	JQ1	Ethanamine	Pomalidomide
**Gefitinib-based PROTAC 3**	276	Gefitinib	1-(2-ethoxyethoxy)pentane	VH032: (S,R,S)-AHPC
**Mcl1 degrader-1**	500	Undefined	hexan-1-amine	Pomalidomide
**MD-224**	50	MI-1242	pent-1-yne	Lenalidomide
**MZ1**	335	JQ1	2-[2-(2-methoxyethoxy)ethoxy]ethanamine	VH032: (S,R,S)-AHPC
**MZP-54**	1652	I-BET726	2-[2-(2-methoxyethoxy)ethoxy]ethanamine	VH032: (S,R,S)-AHPC
**THAL-SNS-032**	799	SNS-032	N-{2-[2-(2-ethoxyethoxy)ethoxy]ethyl}acetamide	Pomalidomide
**VZ185**	22	BI-7273	Pentane	VH101
**ZXH-3-26**	350	JQ1	Pentan-1-amine	Pomalidomide

Table S-2: Ionization states for the PROTAC dataset calculated at pH 7. Not experimentally verified by potentiometry (Sirius T3) due to solubility issues in aqueous environments (water, water-methanol).

PROTACs	Predicted Marvin pK_a (1-14)	Predicted ionization state at pH 7
ACBI1	2.61 (b), 5.39 (b), 7.27 (b), 8.48 (a), 11.05 (a)	32% (N), 62% (+)
ARV-825	4.16 (b), 11.59 (a)	N
BI-0319	2.68 (b), 11.46(a)	N
BI-3663	11.23 (a), 11.84 (a)	N
BI-4206	2.68 (b), 11.46(a)	N
BRD9 degrader-1	7.86 (b), 11.81 (a)	12% (N), 88% (+)
BSJ-03-123	3.19 (b), 7.38 (b), 11.15 (a), 11.79 (a)	29% (N), 70% (+)
*Cis*ACBI1	2.61 (b), 5.39 (b), 7.27 (b), 8.48 (a), 11.05 (a)	32% (N), 62% (+)
*Cis*MZ1	2.6 (b), 4.32 (b)	N
CM11	2.35 (b),2.95 (b)	N
CMP98	2.35 (b),2.95 (b)	N
CRBN-6-5-5-VHL	2.06 (b), 2.79 (b), 11.61 (a)	N
dBET57	4.21(b), 11.61 (a)	N
Gefitinib-based PROTAC 3	2.65 (b), 4.66 (b)	N
Mcl1 degrader-1	2.17(b), 11.61(a)	N
MD-224	9.02 (b), 11.56 (a)	99% (+)
MZ1	2.6 (b), 4.32 (b)	N
MZP-54	3.44 (b), 3.57 (b)	N
THAL-SNS-032	7.11 (b), 8.02 (a), 11.61 (a)	36% (N), 54% (+)
VZ185	2.60 (b), 3.71 (b), 7.99 (b), 11.05 (a)	9% (N), 88% (+)
ZXH-3-26	2.27(b), 3.96(b), 11.61 (a)	N

Table S-3: Experimental solubility values for the PROTAC® dataset.

Molecule	Solubility (mg/mL)	Solubility log S (mol/l)
ACBI1	< 6.80 x10^{-4}	<- 6.14
ARV-825	< 2.90 x10^{-4}	< - 6.5
BI-0319	2.78 x10^{-3}	-5.58
BI-3663	0.01	-5.16
BI-4206	6.06 x10^{-4}	-6.24
BRD9 degrader-1	0.60	-3.18
BSJ-03-123	0.02	-4.75
*Cis*ACBI1	< 8.17 x10^{-4}	< -6.06
*Cis*MZ1	0.05	-4.30
CM11	0.73	-3.20
CMP98	0.39	-3.48
CRBN-6-5-5-VHL	1.19 x10^{-3}	-5.91
dBET57	0.02	-4.52
Gefitinib-based PROTAC 3	1.14 x10^{-3}	-5.91
Mcl1 degrader-1	< 7.93 x10^{-4}	< -6.06
MD-224	< 2.89 x10^{-4}	< -6.64
MZ1	0.04	-4.42
MZP-54	5.28 x10^{-4}	-6.29
THAL-SNS-032	0.05	-4.28
VZ185	0.05	-4.30
ZXH-3-26	0.00	-5.53

Table S-4: In silico log S predictors.

Method	Principle	Source	Structure imput
**● Marvin pH dependent**	Atom-based contribution with 2 correction factors: hydrophobic carbon count and square of molecular weight[28].	https://chemaxon.com/products/marvin	2D
**● Marvin intrinsic**			
**● pkCSM**	Distance-graph based structural signatures[29]	http://biosig.unimelb.edu.au/pkcsm/prediction	
**● Scbdd**	Model based on a similarity engine[30]	http://www.scbdd.com/	
**● AdmetSAR2**	Solvent accessible surface-based[31]	http://lmmd.ecust.edu.cn/admetsar2/	
**● VolSurf pH 7.5**	3D molecular field-based[32]	https://www.moldiscovery.com/software/vsplus/	3D

Table S-5: 2D in silico log P descriptors.

Tool name	log P method	Tool name	log P method	Structure imput
**Marvin**	Chemaxon method: Atom-based (AlogP)	**SwissADME**[35]	iLOGP: Physics-based method.<br>XLOGP: Atom-based.<br>WLOGP: Atom-based.<br>MLOGP: Chemical descriptor based.<br>SILICOS-IT: Hybrid method (Atom based- chemical descriptor based).	2D
**pkCSM**	Distance-based graph structural signatures[22]	**MoKa**	Unknown	
**Scbdd**	Chemical descriptor-based[36]	**ACD Labs**[37]	Fragment group-based:<br>- Classic: Principal of isolating carbons.<br>- GALAS: Similarity-based.<br>- Consensus: Mixed approach.	
**AdmetSAR2**	AlogP[38]	**Molinspiration**	Fragment group-based	
**Volsurf pH 7.5**	3D molecular field-based[25]			3D

Table S-6: Linear correlation of computed log P with experimental solubility.

**In silico predictors**	**log P (scbdd)**	**logD pH 7.4 (scbdd)**	**LogP (ACD)**	**Log D (pH=7.4) (ACD)**	**ALOGP (ADMETS AR2)**	***LOG P n-Oct VolSurf+***
**R square**	0.01	0.01	0.69	0.63	0.51	0.59
**Equation**	Y = -0.09X - 4.56	Y = -0.09X - 4.56	Y = -0.49X - 3.57	Y = -0.43X - 3.47	Y = -0.43X - 2.74	Y = -0.38X - 3.33

**In silico predictors**	**log P (Marvin)**	**miLogP (molinspiration)**	**LogD7.4 MoKa**	**logP MoKa**	**LogD7.5 VolSurf+**	**LOG P n-Oct VolSurf+**
**R square**	0.69	0.63	0.61	0.61	0.53	0.59
**Equation**	Y = -0.55X - 3.83	Y = -0.48X - 3.25	Y = -0.33X - 3.69	Y = -0.40X - 3.31	Y = -0.29X - 3.82	Y = -0.38X - 3.33

**SWISSADME in silico predictors**	***Log Po/w (iLOGP) (Swissadme)***	***Log Po/w (XLOGP3) (Swissadme)***	***Log Po/w (WLOGP) (Swissadme)***	***Log Po/w (MLOGP) (Swissadme)***	***Log Po/w (SILICOS-IT)*** **(Swissadme)**
**R square**	0.01	0.43	0.45	0.28	0.04
**Equation**	Y = -0.07X - 4.39	Y = -0.40X - 3.11	Y = -0.34X - 3.47	Y = -0.34X - 4.89	Y = -0.10X - 4.04

Table S-7. Experimental and calculated solubility for the PROTAC pairs and some of their building blocks. Calculated values discussed in the text are presented in purple (Marvin pH 7).

PROTAC PAIRS	PROTACs and B.Blocks	Exp.LogS (mol/l)	Solubility predictors LogS (mol/l)						
			Admet SAR2	scbdd	pkCSM	Marvin			Volsurf
						Intrinsic	pH 7.4	pH 7	pH 7.5
MZ1-MZP-54	MZ1	-4.42	-3.67	-5.5	-3.13	-6.6	-6.6	-6.6	-9.65
	MZP-54	-6.29	-3.83	-5.67	-2.91	-8.41	-8.41	-8.41	-12.17
	I-BET726	-4.22	-4.14	ND	-4.47	-7.12	-3.87	-4.27	-2.84
	JQ1 (carboxylic acid)	> -2.6	-3.57	-4.71	-3.98	-4.99	-2.34	-2.74	0.05
dBET57-ZXH-3-26	dBET57	-4.52	-3.59	-4.17	-3.99	-7.06	-7.06	-7.06	-6.30
	ZXH-3-26	-5.53	-3.64	-4.70	-3.36	-6.75	-6.75	-6.75	-7.58
	JQ1 (carboxylic acid)	> -2.6	-3.57	-4.71	-3.98	-4.99	-2.34	-2.74	0.05
	PROTAC BET-binding moiety 2	> -2.65	-3.62	-4.82	ND	-4.86	-1.30	-1.7	-0.02
BI-3663-BI-0319/BI-4206	BI-4464	-4.19	-5.75	-5.75	-4.28	-6.52	-5.33	-4.95	-4.68
	BI-3663	-5.16	-3.36	-4.8	-3.06	-7.15	-7.15	-7.15	-8.81
	BI-0319	-5.58	-3.44	-5.16	-2.92	-7.54	-7.54	-7.54	-10.86
	BI-4206	-6.24	-3.44	-5.16	-2.92	-7.54	-7.54	-7.54	-10.95
	Pomalidomide	-4.44	-2.80	-2.44	-2.50	-2.77	-2.77	-2.77	-2.18
	S,R,S-AHPC HCl	-2.68	-3.17	ND	-3.98	-5.02	-4.38	-4.05	-2.99
	S,S,S-AHPC 2HCl	-2.69	-3.17	ND	-3.79	-5.45	-4.81	-4.47	-3.21

Table S-8: Experimental descriptors and calculated TPSA for the explored building blocks. ND: not determined.

MOLECULE	BRlogD	Log k_w^{IAM}	TPSA	ΔLog k_w^{IAM}	Experimental pK_a (2-12)	Ionization state at pH 7
**I-BET726**	0.30	1.88	69.64	2.64	ND	ND
**JQ1 (carboxylic acid)**	0.08	0.80	108.61	1.76	ND	ND
**PROTAC BET-binding moiety 2**	-0.03	0.53	134.91	1.59	ND	ND
**BI-4464**	1.79	1.84	105.68	1.23	ND	ND
**Pomalidomide**	1.41	0.91	111.26	0.64	Not ionizable	Neutral
***S,R,S*-AHPC HCl**	0.56	1.83	136.79	2.34	3.02 (b), 7.59 (b)	+
***S,S,S*-AHPC 2HCl**	0.91	1.86	136.79	2.05	2.79 (b), 7.48 (b)	+

Table S-9: List of the studied PROTACs (alphabetically ordered) with their PROTAC-DATABASE ID and vendor or supplier.

PROTACs	PROTAC-DATABASE-ID	SUPPLIER
**ACBI1**	798	OpnMe
**ARV-825**	329	Medchemexpress
**BI-0319**	1656	OpnMe
**BI-3663**	1655	OpnMe
**BI-4206**		OpnMe
**BRD9 degrader-1**	1187	Chiesi
**BSJ-03-123**	240	Chiesi
**CisACBI1**		OpnMe
**CisMZ1**		OpnMe
**CM11**	998	Tocris
**CMP98**		Tocris
**CRBN-6-5-5-VHL**	176	Tocris
**dBET57**	342	Medchemexpress
**Gefitinib-based PROTAC 3**	276	Chiesi
**Mcl1 degrader-1**	500	Chiesi
**MD-224**	50	Chiesi
**MZ1**	335	OpnMe/Chiesi
**MZP-54**	1652	Medchemexpress
**THAL-SNS-032**	799	Chiesi
**VZ185**	22	OpnMe/Chiesi
**ZXH-3-26**	350	Tocris

Table S-10: Experimental conditions for the explored building blocks. IAM: IAM.PC.DD2 (300 Å, 10 μm, 10 cm x 4.6mm) column. ACN: Acetonitrile. AAB: 20 mM ammonium acetate buffer pH=6.8. MCE: Medchemexpress. C. CURVE: Calibration curve.

MOLECULE	SUPPLIER	C.CURVE	R^2	COLUMN	MOBILE PHASE % (ACN-AAB)	RUN (min)	WAVELENGTH (nm)
**I-BET726**	MCE	y= 611.16x - 0.5843	0.999	IAM	30-70	5	284
**JQ1 (carboxylic acid)**	MCE	y = 451.02x + 10.106	0.989	IAM	30-70	5	255
**PROTAC BET-binding moiety 2**	MCE	y= 465.03x + 0.4861	0.999	IAM	20-80	5	257
**BI-4464**	MCE	y= 302.16x + 0.5924	0.999	IAM	50-50	10	317
**Pomalidomide**	CHIESI	y = 489.98x + 1.095	0.990	IAM	15-85	5	223
***S,R,S*-AHPC HCl**	CHIESI	y = 158.91x- 0.7659	0.998	IAM	25-75	10	269
***S,S,S*-AHPC 2HCl**	CHIESI	y= 253.94x - 0.5771	1.000	IAM	25-75	10	269

Table S-11: Experimental chromatographic conditions for PROTAC® solubility measurements. IAM: IAM.PC.DD2 (300 Å, 10 µm, 10 cm x 4.6mm) column. ACN: Acetonitrile. AAB: 20 mM ammonium acetate buffer pH=6.8.

MOLECULE	COLUMN	MOBILE PHASE % (ACN-AAB)	RUN-TIME	WAVELENGTH (nm)
ACBI1	IAM	50-50	5	266
ARV-825	IAM	40-60	8	260
BI-0319	IAM	40-60	5	281
BI-3663	IAM	50-50	5	318
BI-4206	IAM	40-60	5	281
BRD9 degrader-1	IAM	40-60	5	234
BSJ-03-123	IAM	50-50	5	354
*Cis*ACBI1	IAM	50-50	5	266
*Cis*MZ1	IAM	40-60	7	261
CM11	IAM	30-70	5	273
CMP98	IAM	30-70	5	273
CRBN-6-5-5-VHL	IAM	40-60	7	271
dBET57	IAM	40-60	5	256
Gefitinib-based PROTAC 3	IAM	50-50	5	251
Mcl1 degrader-1	IAM	50-50	5	234
MD-224	IAM	60-40	5	257
MZ1	IAM	40-60	7	261
MZP-54	IAM	40-60	5	284
THAL-SNS-032	IAM	40-60	5	270
VZ185	IAM	40-60	9	203
ZXH-3-26	IAM	30-70	15	254

Table S-12: Calibration curves for the PROTAC dataset.

MOLECULE	CALIBRATION CURVE	R^2
ACBI1	y= 243.81x - 0.0665	1.000
ARV-825	y = 286.63x -0.1357	0.983
BI-0319	y= 173.3x - 0.2069	0.996
BI-3663	y= 173.3x - 0.2069	0.996
BI-4206	y= 194.97x - 0.0801	0.996
BRD9 degrader-1	y= 278.99x - 1.6035	1.000
BSJ-03-123	y= 148.98x - 0.0549	0.999
*Cis*ACBI1	y= 281.25x - 0.0696	1.000
*Cis*MZ1	y = 247.04x - 0.7473	0.995
CM11	y= 78.792x - 1.1708	0.998
CMP98	y= 108.92x - 0.6903	0.997
CRBN-6-5-5-VHL	y= 91.203x - 0.0957	0.993
dBET57	y= 284.25x - 0.0524	0.999
Gefitinib-based PROTAC 3	y= 173.98x-0.1394	0.995
Mcl1 degrader-1	y= 306.1x -0.0639	0.999
MD-224	y= 331.17x-0.0542	1.000
MZ1	y = 546.12X -0.1407	0.999
MZP-54	y= 243.29x - 0.0799	0.998
THAL-SNS-032	y= 71.561x-0.0022	1.000
VZ185	y= 142.99x - 0.6214	0.998
ZXH-3-26	y= 396.78x - 0.0469	1.000

Figure S-1: Structures of the 21 PROTACs ® considered by the study

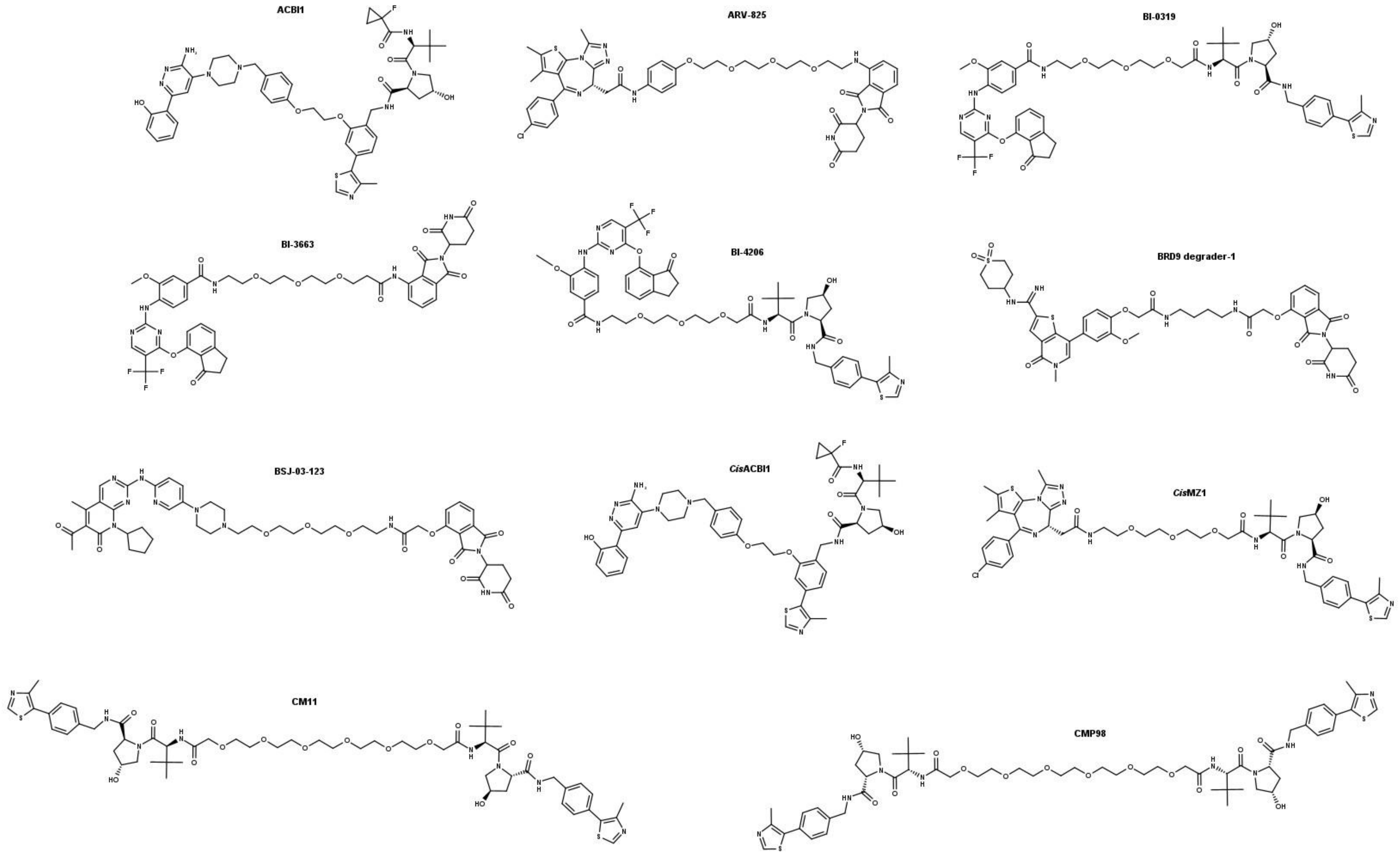

CRBN-6-5-5-VHL

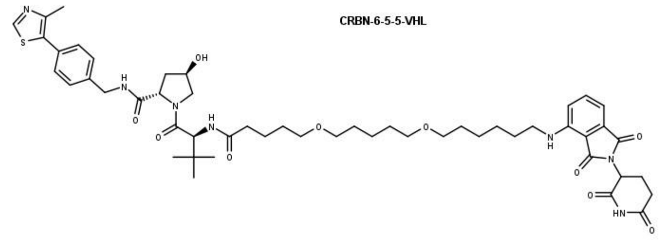

DBET57

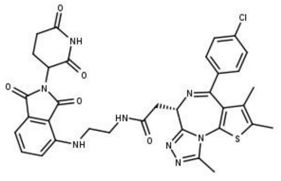

Gefitinib-based PROTAC 3

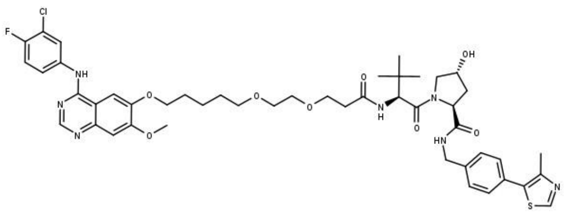

Mcl1 degrader-1

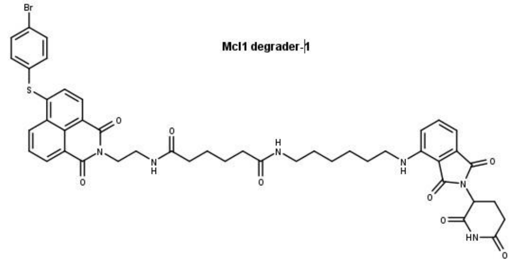

MD-224

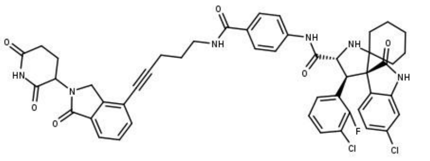

MZ1

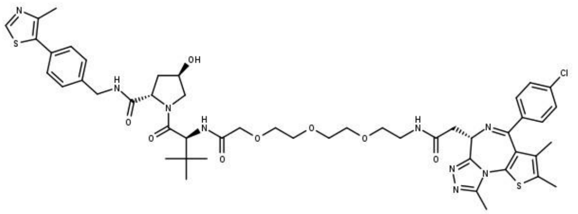

MZP-54

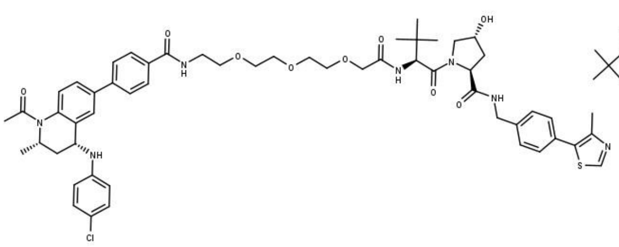

THAL-SNS-032

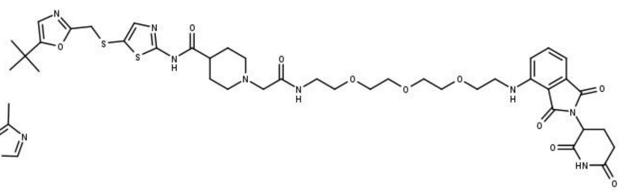

VZ185

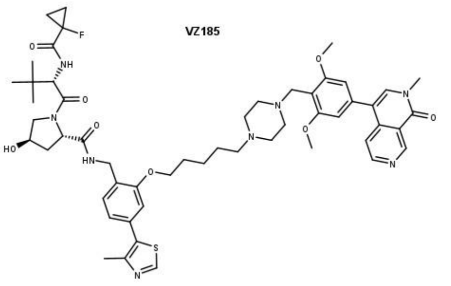

ZXH-3-26

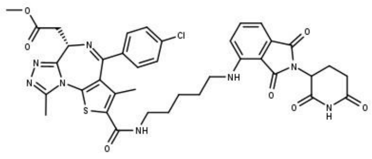

Figure S-2: Experimental solubility vs Δlog k_w^{IAM} for the PROTAC® dataset.

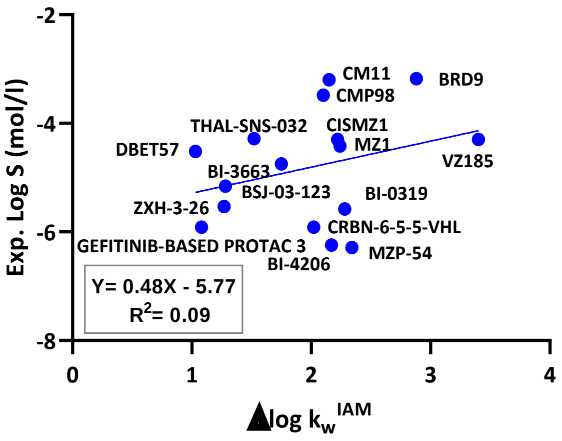

Figure S-3: Δlog k_w^{IAM} vs TPSA for the PROTAC® dataset.

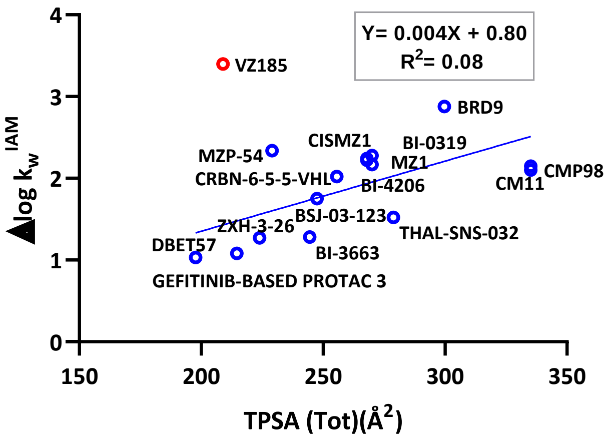

Figure S-4: Experimental solubility vs MW for the PROTAC® dataset.

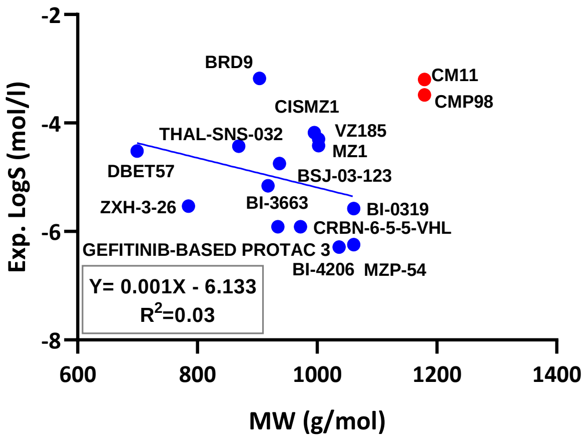

Figure S-5: PROTAC® solubility distribution (log k_w^{IAM}, BRlogD and TPSA) colored by the GSK classification: low (<30 µM), intermediate (30–200 µM) or highly soluble molecules (>200 µM). PROTACs® without an accurate solubility (red values from Table 1) were included in the low solubility class.

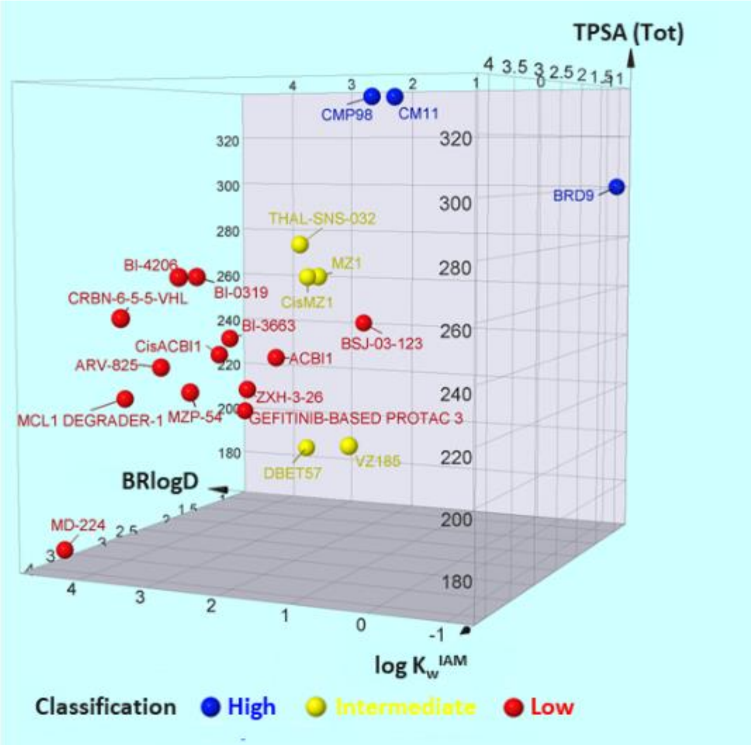

Figure S-6: Weka models. A) Random Tree B) Random Forest.

A) Random Tree

Correctly Classified Instances	13 (87%)
Incorrectly Classified Instances	2 (13%)
Total Number of Instances	15

TP Rate	FP Rate	Precision	Recall	F-Measure	Matthews Correlation Coefficient (MCC)	Class
0.667	0.083	**0.667**	0.667	0.667	0.583	high
0.800	0.100	**0.800**	0.800	0.800	0.700	intermediate
1.000	0.000	**1.000**	1.000	1.000	1.000	low

Confusion Matrix				
a	b	c	Classified as	
2	1	0	a =	high
1	4	0	b =	intermediate
0	0	7	c =	low

B) Random Forest

Correctly Classified Instances	13 (87%)
Incorrectly Classified Instances	2 (13%)
Total Number of Instances	15

TP Rate	FP Rate	Precision	Recall	F-Measure	Matthews Correlation Coefficient (MCC)	Class
0.667	0.083	**0.667**	0.667	0.667	0.583	high
0.800	0.100	**0.800**	0.800	0.800	0.700	intermediate
1.000	0.000	**1.000**	1.000	1.000	1.000	low

Confusion Matrix				
a	b	c	Classified as	
2	1	0	a =	high
1	4	0	b =	intermediate
0	0	7	c =	low

Figure S-7: HPLC traces (IAM column).

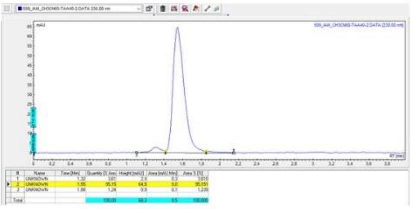

BRD9 degrader-1

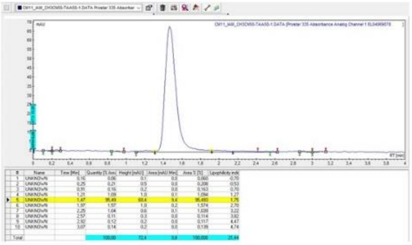

CM11

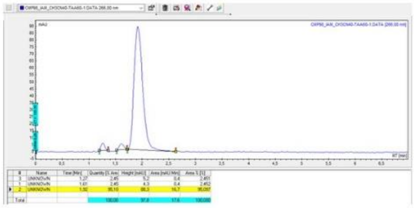

CMP98

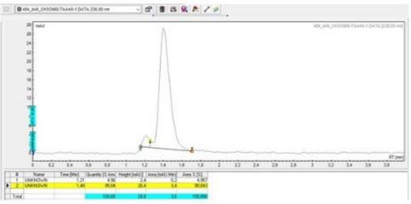

THAL-SNS-032

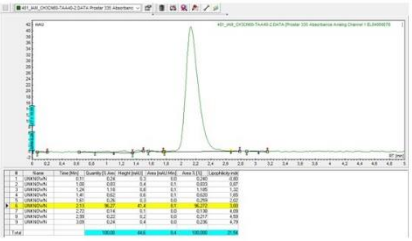

VZ185

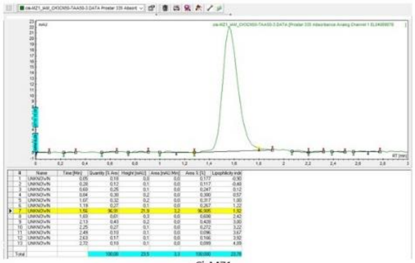

*Cis*MZ1

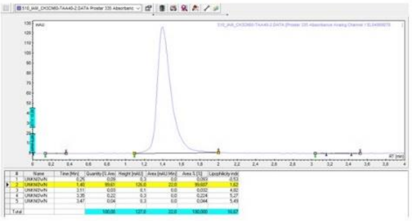

MZ1

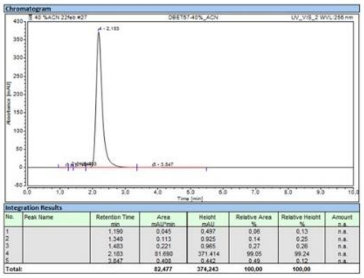

dBET57

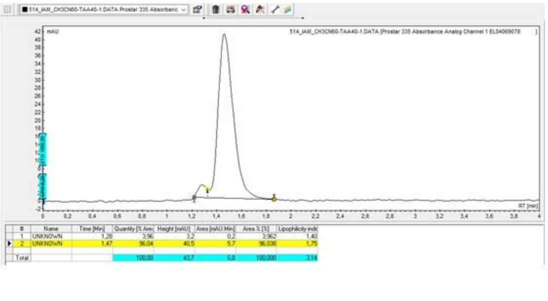

BSJ-03-123

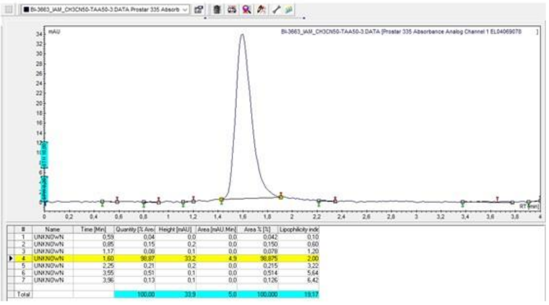

BI-3663

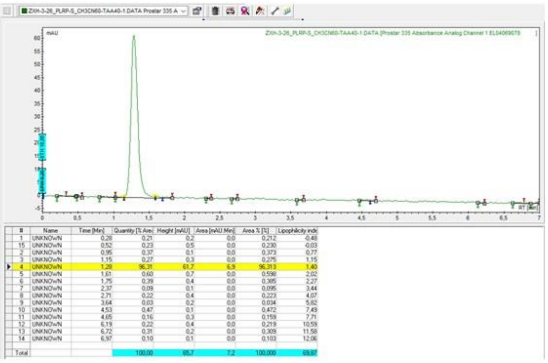

ZXH-3-26

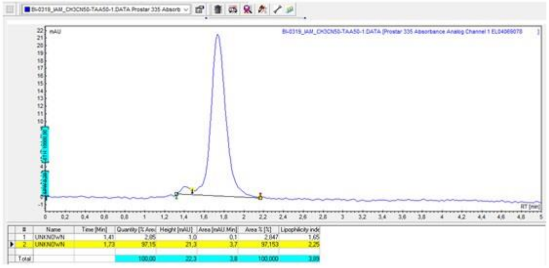

BI-0319

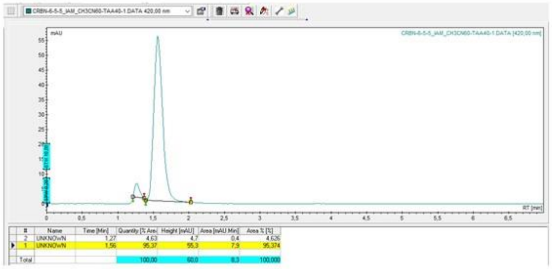

CRBN-6-5-5-VHL

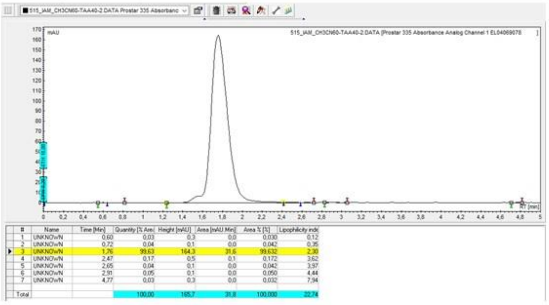

Gefitinib-based PROTAC 3

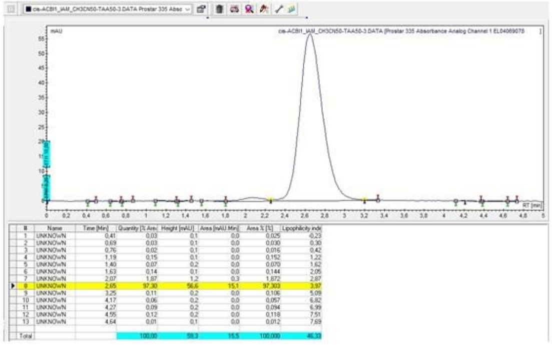

*Cis*ACBI1

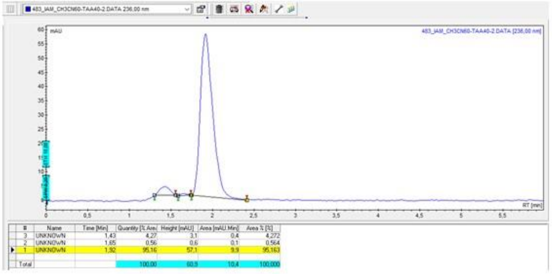

Mcl1 degrader-1

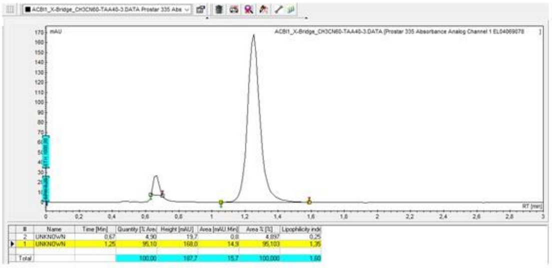

ACBI1

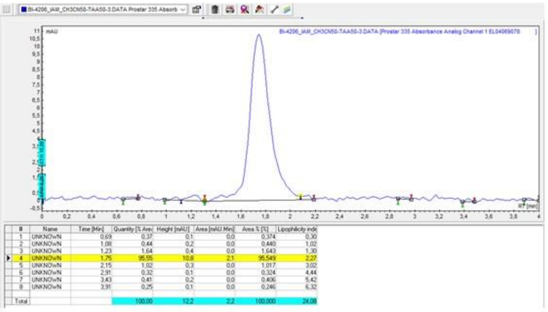

BI-4206

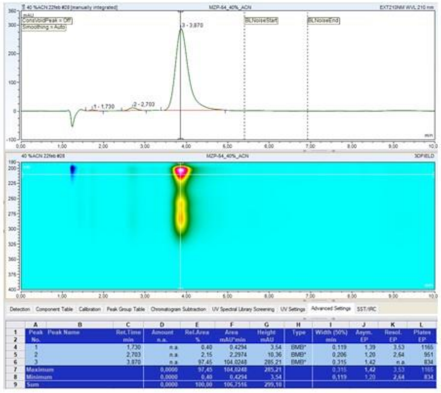

MZP-54

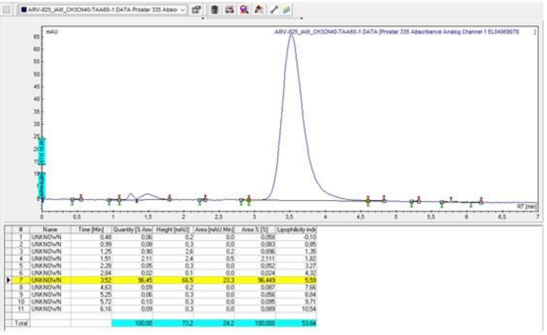

ARV-825

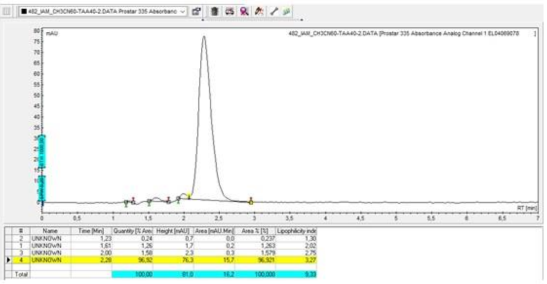

MD-224
